# Applied Mathematics of Ray Tracing and Perspective Projection in Fiducial-Based Registration of X-Ray Images

**DOI:** 10.7759/cureus.7904

**Published:** 2020-04-30

**Authors:** Mark Sedrak, Armando L Alaminos-Bouza

**Affiliations:** 1 Neurosurgery, Northern California Kaiser Permanente, Redwood City, USA; 2 Medical Physics, MEVIS Informática Médica Ltda., São Paulo, BRA

**Keywords:** 2d3d, x-ray analysis, ray tracing, perspective projection, biplanar analysis, x-ray, fluoroscopy, cartesian

## Abstract

Ray tracing (RT) and perspective projection (PP) using fiducial-based registration can be used to determine points of interest in biplanar X-ray imaging. We sought to investigate the implementation of these techniques as they pertain to X-ray imaging geometry. The mathematical solutions are presented and then implemented in a phantom and actual case with numerical tables and imaging. The X-ray imaging is treated like a Cartesian system in millimeters (mm) with a standard frame-based stereotactic system. In this space, the point source is the X-ray emitter (focal spot), the plane is the X-ray detector, and fiducials are in between the source and plane. In a phantom case, RT was able to predict locations of fiducials after moving the point source. Also, a scaled PP matrix could be used to determine imaging geometry, which could then be used in RT. Automated identification of spherical fiducials in 3D was possible using a center of mass computation with average Euclidean error relative to manual measurement of 0.23 mm. For PP, RT projection or a combinatorial approach could be used to facilitate matching 3D to 2D points. Despite being used herein for deep brain stimulation (DBS), utilization of this kind of imaging analysis has wide medical and non-medical applications.

## Introduction

In 1895, Wilhelm Roentgen discovered X-rays, which subsequently had an important impact in medicine for over 100 years [1]. As a major diagnostic tool, X-rays revolutionized medicine by empowering clinicians to diagnose various conditions such as pneumonia, bony fractures, and dental cavities. Despite the long history of X-ray use, developments primarily have included advances in speed, quality, and, more recently, digitization. Computational X-ray analysis has not been the standard despite the wide use of X-rays around the world. Rather, advancements in computed tomography (CT) and magnetic resonance imaging (MRI) have been utilized for 3D (three dimension) localization. Nevertheless, X-rays represent a simple, low cost, low radiation dose technique for which the utility can be optimized.

With the addition of more than a single X-ray plane, stereotactic intraoperative location (STiL) during deep brain stimulation (DBS) surgeries has been demonstrated to provide accurate 3D positions using ray tracing (RT) and perspective projection (PP) [2]. RT and PP assume a Cartesian-based system where from a point source (X-ray emitter focal spot), rays are created through objects (fiducials) to a detector plane (X-ray cartridge), which are then presented on a display plane (the X-ray image). Computationally, RT requires knowledge of the entire imaging geometry, whereas PP requires knowledge of the 3D points and their associated projection points in 2D (two dimension). Using these techniques, RT and PP represent important tools that offer critical analysis of X-rays.

In this paper, we discuss the mathematics involved with RT and PP as well as provide numerical samples and imaging of each. We analyze a phantom case, a real case, explore automations and error propagation. These methods effectively compute 3D to 2D (3D-2D) and, for STiL, 2D to 3D (2D-3D). We explore these techniques using point analysis, but similar techniques can be expanded using volumes.

## Technical report

In stereotactic neurosurgery, a Cartesian coordinate system can be generated using anatomical structures in the brain or using a frame-based apparatus. Anatomical points include the anterior commissure (AC), posterior commissure (PC), and a midline structure to generate the coordinate system, generally from the middle of AC-PC (MidACPC). Frame-based coordinate systems typically use an N-localizer apparatus calibrated on a CT or MRI [3-5]. Generally, these coordinate systems are considered linear and are scaled in millimeters. In the following, we implement mathematical solutions for point-based registration from 3D (x, y, z) to 2D (U, V) using RT and PP. These techniques assume that there are no geometrical distortions (scattering or diffraction), that the focal spot of the X-ray emitter is a point source, that the X-ray detector can be treated like a plane, and that the center of spheres or pin tips/bases can be treated as point objects in the system (fiducials). The centers of spheres are used in the majority of cases as the center is geometrically ideal, but we also utilize pin tips and pin bases in addition to other stable objects when available, which depends on the X-ray exposure. All objects are mathematically utilized as points. For the display plane (X-ray image), pixel spacing may be present from DICOM metadata, but this is often not present in fluoroscopic images. In some instances, calibration may be needed to determine this scaling and ensure no inhomogeneity in the image. Also, to maintain a single frame-based coordinate system, the subsequent tables utilize CRW/BRWLF (Cosman-Roberts-Wells/Brown-Roberts-Wells Localizer Frame, Radionics CRW Stereotactic System, Integra LifeSciences Corporation, Plainsboro, New Jersey). Fiducial positions were calculated using CT with the incorporation of important points fused from MRI in iPlan 3.0 Stereotaxy (Brainlab Inc, Feldkirchen, Germany). Also, the numerical tables utilize AP (Antero-Posterior), LAT (Lateral), VERT (Vertical) rather than x, y, z. Finally, many of the solutions provided assume biplanar imaging (two views or poses), but the same mathematical solutions can allow many more.

Background mathematical solutions

**Figure 1:**
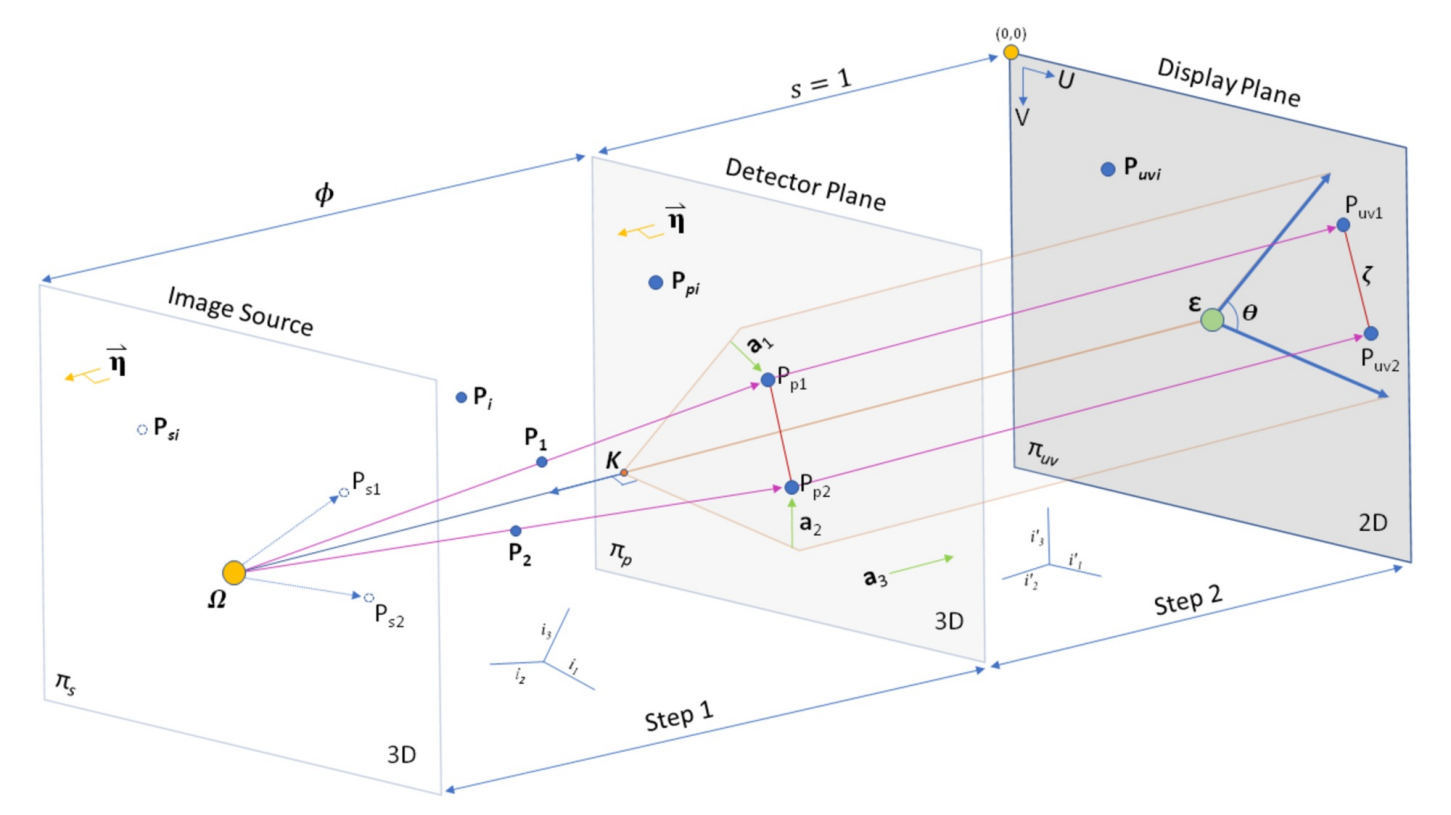
Geometry of X-ray Imaging from 3D to 2D. The imaging takes the form of a Cartesian system based on stereotactic space. The image source contains a plane that is parallel to the detector plane with a point source at X-ray emitter (focal spot). The focal spot generates rays (or lines) that intersect objects (fiducials) and then intersect the detector plane. The detector plane is then converted to the display plane, which is what is seen in the image. This conversion effectively transforms 3D space to 2D in Ray Tracing (RT) and Perspective Projection (PP) via Step 1 and Step 2. Keeping the entire relationship in millimeters with equal scaling (s = 1) between detector plane and display plane allows visual inspection of the relationships, which demonstrates that the scalar distance between projection objects should be the same between the detector plane and display planes. The normal of the detector plane is parallel to the normal of a plane that intersects the point source. For both RT and PP, a normalization process occurs making 3D space orthogonal to the display plane. Of note, the initial coordinate system, which could be the stereotactic coordinate system, is not Coherent On-Axis (COA) to the detector plane. In PP, a projection matrix can be generated using knowledge of 3D points (fiducials) combined with their associated position on the display plane. A non-skew PP matrix signifies that the angle (theta) is 90 degrees. Coordinates are thought of as 3D (x,y,z) in Step 1, then in Step 2 are still 3D for RT (x,y,z) or PP (u,v,t). Once images are projected on the display plane, they are 2D (U, V). The PP matrix can be used to determine the location of the point source. From the intrinsic matrix of PP, the distance between the image source and the detector plane (focal length) can be calculated, thereby allowing calculation of the detector plane location. The point on the detector plane that is closest to the focal spot can be utilized to calculate the principal point on the display plane. Finally, in the display plane, we use the top left corner of the image assigned as (0,0). $\eta = Normal\: of\: Plane$ $\Omega \: = \: P_{0} = Point Source = X- \: ray \: emitter \: (Focal \: Spot)$ $\kappa\: = \: Closest \: Point \: in \: detector \: plane \: to \: source$ $\epsilon\: = \: Principal \: Point \: on \: display \: plane$ $\Pi_{s} \: = \: image  \: source  \: plane$ $\Pi_{p} \: = \: Detector \: plane$ $\Pi_{uv} \: = \: Display \: plane$ $P_{1} \: and \: P_{2} \: = \: fiducials  \: in  \: space$ $P_{p1} \: and \: P_{p2} \: = \: points \: of \: intersection \: on \: detector \: plane$ $P_{uv1} \: and \: P_{uv2} \: = \: points \: of \: intersection \: on \: display \: plane \: = (U_{p1}, V_{p1}) \: and \: (U_{p2}, V_{p2})$ $P_{i} \: = \: a\: point \: in \: between \: source \: and \: detector \: plane$ $P_{si} \: = \: a\: point \: on \: image \: source \: plane$ $P_{pi} \: = \: a\: point \: on \: detector \: plane$ $P_{uvi} \: = \: a\: point \: on \: display \: plane$ $i_{1}, \: i_{2}, \: i_{3} \: represent \: stereotactic \: coordinate \: axis\: which \: is \: normalized\: to \:  i'_{1}, \: i'_{2}, \: i'_{3}, \: prior\: to \: projection \: on \: display \: plane$ $x, \: y, \: z \: = \: 3D\: coordinates$ $u, \: v, \:t \: = \: 3D \: coordinates\: in \: PP$ $U \: = \: Horizontal \: 2D \: coordinates \: on \: display \: plane$ $V \: = \: Vertical \: 2D \: coordinates \: on \: display \: plane$ $\zeta \: = \: scalar \: distance \: between \: projection \: points$ $\: \theta \: = \: determines \: skew \: angle$ $\: \phi \: = \: focal \: length \: = \: Source-\: image- \: distance \: (SID)$ $\: s  \: = \: scaling \: between \: detector \: plane \: and \: display \: plane$ $\: \textbf{a}_{1}  \: = \: first \: vector \: component \: of \: perspective \: projection \: matrix$ $\: \textbf{a}_{2}  \: = \: second \: vector \: component \: of \: perspective \: projection \: matrix$ $\: \textbf{a}_{3}  \: = \: third \: vector \: component \: of \: perspective \: projection \: matrix$

In RT, the geometry of the point source (focal spot size) (P_0_) to a fiducial (P_1 _or P_2_) forms a ray that intersects a detector (P_p1_ or P_p2_) plane, which is seen on a display (P_uv1_ or P_uv2_) plane (Figure 1) [6]. If the stereotactic coordinate system utilized contains an axis orthogonal to the detector plane, the system is coherent on-axis (COA). Having a COA system makes the determination of display points more simple, such that the two variable coordinates are translated, rotated and scaled to the display plane. The display plane can also be rotated separately. If the detector plane is not COA, then a change of basis transformation is needed to normalize an axis. For example, in a COA antero-posterior (AP) view, the AP value of all plane intersections with the AP plane is the same, which allows straightforward utilization of the other dimensions, lateral (LAT) and vertical (VERT), where the intersections occur. However, a system that is not COA yields 3D coordinates for which all values have variation, requiring a normalization prior to projection to the display plane.

The X-ray detector can be considered as a mathematical plane (equation 1). The X-ray emitter then functions as a point source that generates rays which intersect fiducials, generating lines in 3D parametric form (equations 2, 3, 4). When these lines intersect the X-ray detector (Step 1, Figure 1), there are the points in the detector plane (equations 5, 6, 7). Because the display plane would vary in presentation, added translation/rotation is required for pixel location, which can be computed in matrix form incorporating this 2D rotation and translation (equation 8). Here, the 2D presentation of the planar image expects a dimension of the 3D intersection to be a constant (COA). When not COA, the normalization procedure (Step 2, Figure 1) is simplified by the use of unit vectors (equation 9) and dot-products between the two coordinate systems thereby identifying the matrix components.

$$Ax + By +Cz + D = 0\tag{1}$$

$$x = x_{1} + a_{1}\cdot r_{1}\tag{2}$$

$$y = y_{1} + b_{1}\cdot r_{1}\tag{3}$$

$$z = z_{1} + c_{1}\cdot r_{1}\tag{4}$$

$$x = x_{1} - \frac{a_{1}(Ax_{1}+By_{1}+Cz_{1}+D)}{Aa_{1}+Bb_{1}+Cc_{1}}\tag{5}$$

$$y = y_{1} - \frac{b_{1}(Ax_{1}+By_{1}+Cz_{1}+D)}{Aa_{1}+Bb_{1}+Cc_{1}}\tag{6}$$

$$z = z_{1} - \frac{c_{1}(Ax_{1}+By_{1}+Cz_{1}+D)}{Aa_{1}+Bb_{1}+Cc_{1}}\tag{7}$$

$$\begin{bmatrix} U'\\ V' \\ 1 \end{bmatrix} = \begin{bmatrix} r_{11} & r_{12} & t_{1}\\ r_{21} & r_{22}& t_{2} \\ 0 & 0 & 1 \end{bmatrix} \cdot \begin{bmatrix} U\\ V \\ 1 \end{bmatrix}\tag{8}$$

$$\begin{bmatrix} x_{1}'\\ y_{2}'\\ z_{3}' \end{bmatrix} = \begin{bmatrix} i_{1}'\cdot i_{1} & i_{1}'\cdot i_{2} & i_{1}'\cdot i_{3}\\ i_{2}'\cdot i_{1} & i_{2}'\cdot i_{2} & i_{2}'\cdot i_{3}\\ i_{3}'\cdot i_{1} & i_{3}'\cdot i_{2} & i_{3}'\cdot i_{3} \end{bmatrix} \cdot \begin{bmatrix} x_{1}\\ y_{2}\\ z_{3} \end{bmatrix}\tag{9}$$

In perspective projection (PP) a 3D point in space is associated with a 2D point on the display plane (equation 10) [7]. Importantly, a 2D point is generated (Step 2, Figure 1) by dividing the third component (t) with u or v (equations 11-12). A 4x3 conversion matrix is used wherein 11 of the 12 elements are unknowns initially. The three columns represent the projection matrix components (equations 13-15). Each of these three column components has four numerical values, but the last value (C_43_) is scaled to 1 in this homogenous formulation (equations 16-19). The system is then arranged as a set of overdetermined equations generally, such that each instance of 3D-2D points generates two equations (equation 20), one for U and another for V. Therefore, 5.5 3D-2D points are needed to solve the 11 matrix elements, but generally this system would be solved via a least squares formulation to optimize the matrix and minimize errors. Lastly, the complete PP matrix is 4x3 (equation 21), but will be subsequently utilized as its transpose in 3x4 (equation 22).

$$\left ( x,y,z,1 \right )\cdot C = (u,v,t)\tag{10}$$

$$U = u/t\tag{11}$$

$$V = v/t\tag{12}$$

$$u = (x,y,z,1)\cdot C_{1}\tag{13}$$

$$v = (x,y,z,1)\cdot C_{2}\tag{14}$$

$$t = (x,y,z,1)\cdot C_{3}\tag{15}$$

$$C_{1} = \begin{bmatrix} C_{11}\\ C_{21}\\ C_{31}\\ C_{41} \end{bmatrix}\tag{16}$$

$$C_{2} = \begin{bmatrix} C_{12}\\ C_{22}\\ C_{32}\\ C_{42} \end{bmatrix}\tag{17}$$

$$C_{3} = \begin{bmatrix} C_{13}\\ C_{23}\\ C_{33}\\ C_{43} \end{bmatrix}\tag{18}$$

$$C_{43} = 1\tag{19}$$

$$\begin{bmatrix} x_{1} & y_{1} & z_{1} & 1 & 0 & 0 & 0 & 0 & -U_{1}x_{1} & -U_{1}y_{1} & -U_{1}z_{1}\\ 0 & 0 & 0 & 0 &x_{1} & y_{1} & z_{1} & 1 & -V_{1}x_{1} & -V_{1}y_{1} & -V_{1}z_{1}\\ \vdots & & & & & & & & & & \vdots\\ x_{n} & y_{n} & z_{n} & 1 & 0 & 0 & 0 & 0 & -U_{n}x_{n} & -U_{n}y_{n} & -U_{n}z_{n}\\ 0 & 0 & 0 & 0 &x_{n} & y_{n} & z_{n} & 1 & -V_{n}x_{n} & -V_{n}y_{n} & -V_{n}z_{n} \end{bmatrix} \cdot \begin{bmatrix} C_{11}\\ C_{21}\\ \vdots\\ C_{33} \end{bmatrix} = \begin{bmatrix} U_{1}\\ V_{1}\\ \vdots\\ U_{n}\\ V_{n} \end{bmatrix}\tag{20}$$

$$C = \begin{bmatrix} C_{11} & C_{12} & C_{13} \\ C_{21} & C_{22} & C_{23}\\ C_{31} & C_{32} & C_{33}\\ C_{41} & C_{42} & C_{43} \end{bmatrix}\tag{21}$$

$$C^{T} = \begin{bmatrix} a_{11} & a_{21} & a_{31} & a_{41}\\ a_{12} & a_{22} & a_{32} & a_{42} \\ a_{13} & a_{23} & a_{33} & a_{43} \end{bmatrix}\tag{22}$$

Interpretation of the PP model is generally widespread in computer vision [7]. Next, we utilize this technique to gain insight into the geometric interpretation of the X-ray system. The essential elements of the complete PP matrix, here now interpreted as 3x4, include a 3x3 intrinsic parameter and the extrinsic parameters include a 3x3 rotation and a 3x1 translation (equation 23). The components of the intrinsic parameter are important to note, which include scalar dimensions, shear, and translation (equation 24). The five intrinsic components are alpha and beta (which should be equal), theta, and the principal point (U_0_, V_0_). Of note, a skew angle is computed as the absolute value of theta minus pi/2. Again, to calculate these values for geometric interpretation, the scale of the imaging system (detector plane and display plane) should be the same or undergo a conversion. For example, the imaging plane may generate uncalibrated pixels and the stereotactic frame (or anatomic frame) is in millimeters. If the U and V coordinates on the imaging plane are converted to a millimeter system, interpretation of the components of the intrinsic parameters is facilitated. Notably, this scaling is not required for STiL using PP. Next, the vector components (equations 25-27) of the PP matrix are utilized to calculate components of these intrinsic parameters (equations 28-31). Of note, the expected result of equation 31 is 0, as in an ideal setting the imaging system would be non-skew (theta = pi/2 (approximately 1.57) radians or 90 degrees). The next elements calculated are utilized to generate the point source and the imaging plane. To formulate relevant information, the negative inverse (-a^-1^) of the vector component (3x3) of the PP matrix is performed and multiplied by a translation (equations 32-25). Visually, we see from Figure 1 that a 2D point on the display plane can be projected back to a plane that intersects the point source and is parallel to the detector plane. First, by using the translation component of the original PP matrix (b) and multiplying that with the negative inverse of the vector component, the point source (Omega) is then determined (equation 36). Picking two more unique 2D points (c, d) in the display plane generates two more points in the plane that intersect the point source (equations 37-38). Using these three points in the image source plane, two vectors can be created (equations 39-40). Forward or reverse cross-products of these vectors (equation 41-42) yields the normal of the image source plane, which is parallel to the detector plane (equation 43). This result may be expressed as a plane equation wherein the last element (sigma) is not yet known (equation 44). However, sigma can be determined using components of the intrinsic matrix for focal length, which in X-ray imaging is equivalent to the Source Image Distance (SID) (equations 45-46). It is possible, and even likely, under many circumstances that the imaging system may be slightly skew, so taking the average of each scalar component (alpha and beta) may yield a good approximation (equation 47). Furthermore, the relationship between focal length, the point source, and the detector plane tie all these elements together (equation 48). If the focal length is known, then one can readily calculate the missing component (sigma) of the detector plane equation.

$$C = K^{3,3} \begin{bmatrix} R^{3,3} & \textbf{b}^{3,1} \end{bmatrix}\tag{23}$$

$$K = \begin{bmatrix} \alpha & -\alpha cot\theta & U_{0}\\ 0 & \beta /sin\theta & V_{0}\\ 0 & 0 & 1 \end{bmatrix}\tag{24}$$

$$\textbf{a}_{1} = \begin{bmatrix} a_{11} & a_{21} & a_{31} \end{bmatrix}\tag{25}$$

$$\textbf{a}_{2} = \begin{bmatrix} a_{12} & a_{22} & a_{32} \end{bmatrix}\tag{26}$$

$$\textbf{a}_{3} = \begin{bmatrix} a_{13} & a_{23} & a_{33} \end{bmatrix}\tag{27}$$

$$\rho = \pm {1}/\left | \textbf{a}_{3}\right |{}\tag{28}$$

$$U_{0}=\rho ^2(\boldsymbol{a}_{1}\cdot \boldsymbol{a}_{3})\tag{29}$$

$$V_{0}=\rho ^2(\boldsymbol{a}_{2}\cdot \boldsymbol{a}_{3})\tag{30}$$

$$cos\theta = (\boldsymbol{a}_{1} \times \boldsymbol{a}_{3})\cdot (\boldsymbol{a}_{2} \times \boldsymbol{a}_{3}) /\left | \boldsymbol{a}_{1} \times \boldsymbol{a}_{3} \right |\cdot \left | \boldsymbol{a}_{2} \times \boldsymbol{a}_{3} \right |\tag{31}$$

$$-a^{-1} = \begin{bmatrix} -a_{11} & -a_{21} & -a_{31}\\ -a_{12} & -a_{22} & -a_{32} \\ -a_{13} & -a_{23} & -a_{33} \end{bmatrix}^{-1}\tag{32}$$

$$\textbf{b} = \begin{bmatrix} a_{41}\\ a_{42}\\ a_{43} \end{bmatrix}\tag{33}$$

$$\textbf{c} = \begin{bmatrix} U_{i}\\ V_{i}\\ 1 \end{bmatrix}\tag{34}$$

$$\textbf{d} = \begin{bmatrix} U_{n}\\ V_{n}\\ 1 \end{bmatrix}\tag{35}$$

$$\Omega = -a^{-1} \cdot \textbf{b}\tag{36}$$

$$\Delta = -a^{-1} \cdot \textbf{c}\tag{37}$$

$$\Psi = -a^{-1} \cdot \textbf{d}\tag{38}$$

$$\vec{v_{1}} = \Delta -\Omega\tag{39}$$

$$\vec{v_{2}} = \Psi -\Omega\tag{40}$$

$$\vec{n_{p}} = \vec{v_{1}} \times \vec{v_{2}}\tag{41}$$

$$\vec{n_{p}} = \vec{v_{2}} \times \vec{v_{1}}\tag{42}$$

$$\vec{n_{p}} = {(\lambda ,\mu ,\kappa)}\tag{43}$$

$$\lambda x + \mu y + \kappa z + \sigma = 0\tag{44}$$

$$\alpha = \rho^2\left | \textbf{a}_{1}\times \textbf{a}_{3} \right | sin\theta\tag{45}$$

$$\beta = \rho^2\left | \textbf{a}_{2}\times \textbf{a}_{3} \right | sin\theta\tag{46}$$

$$\phi \approx (\alpha +\beta )/2\tag{47}$$

$$\phi = \left | \lambda x_{0} + \mu y_{0} + \kappa z_{0} + \sigma \right |/\sqrt{\lambda^2 + \mu^2 + \kappa^2}\tag{48}$$

Further evaluation of the geometry reveals that the relationship required for solving the sigma of the detector plane equation can be alternatively solved using a quadratic solution (Figure 1) as explained below. First, consider two sets of parametric equations of 3D lines that emerge from the point source through 3D points (equations 49-54). These lines then intersect the plane at some points (equations 55-60). Interestingly, PP allows one to determine the 2D scalar distance, which is a Euclidean distance, on the display plane using a projection of two 3D (we used {1,1,1} and {100, 100, 100}) points using equation 10. This scalar distance is equal to zeta in equation 61 and could alternatively be measured manually on the display plane. The final formation when squared reveals an equation that has a single unknown, sigma (equation 62). Finally, sigma can be solved here via the quadratic method (equations 63-64).

$$a_{1} = x_{1} - x_{0}\tag{49}$$

$$b_{1} = y_{1} - y_{0}\tag{50}$$

$$c_{1} = z_{1} - z_{0}\tag{51}$$

$$a_{2} = x_{2} - x_{0}\tag{52}$$

$$b_{2} = y_{2} - y_{0}\tag{53}$$

$$c_{2} = z_{2} - z_{0}\tag{54}$$

$$x_{p1} = x_{1} - ( a_{1}*( \lambda*x_{1} + \mu*y_{1} + \kappa*z_{1} + \sigma)/ (\lambda*a_{1} + \mu*b_{1} + \kappa*c_{1}) )\tag{55}$$

$$y_{p1} = y_{1} - ( b_{1}*( \lambda*x_{1} + \mu*y_{1} + \kappa*z_{1} + \sigma)/ (\lambda*a_{1} + \mu*b_{1} + \kappa*c_{1}) )\tag{56}$$

$$z_{p1} = z_{1} - ( c_{1}*( \lambda*x_{1} + \mu*y_{1} + \kappa*z_{1} + \sigma)/ (\lambda*a_{1} + \mu*b_{1} + \kappa*c_{1}) )\tag{57}$$

$$x_{p2} = x_{2} - ( a_{2}*( \lambda*x_{2} + \mu*y_{2} + \kappa*z_{2} + \sigma)/ (\lambda*a_{2} + \mu*b_{2} + \kappa*c_{2}) )\tag{58}$$

$$y_{p2} = y_{2} - ( b_{2}*( \lambda*x_{2} + \mu*y_{2} + \kappa*z_{2} + \sigma)/ (\lambda*a_{2} + \mu*b_{2} + \kappa*c_{2}) )\tag{59}$$

$$z_{p2} = z_{2} - ( c_{2}*( \lambda*x_{2}+ \mu*y_{2} + \kappa*z_{2} + \sigma)/ (\lambda*a_{2} + \mu*b_{2} + \kappa*c_{2}) )\tag{60}$$

$$\zeta_{12} = \sqrt( (x_{p1} - x_{p2})^2 + (y_{p1} - y_{p2})^2 + (z_{p1} - z_{p2})^2) = \sqrt( (U_{p1} - U_{p2})^2 + (V_{p1} - V_{p2})^2 )\tag{61}$$

$$\zeta_{12}^2 = ( (x_{p1} - x_{p2})^2 + (y_{p1} - y_{p2})^2 + (z_{p1} - z_{p2})^2) \tag{62}$$

$$0 = i\sigma^2 + j\sigma + k\tag{63}$$

$$\sigma = ( -j \pm \sqrt(j^2 - 4ik)/2i)\tag{64}$$

Fiducial matching from 3D to 2D

A time-consuming and potentially error-prone task is matching 3D to 2D fiducials for PP. One method is to utilize knowledge of the geometric setup and perform RT-assisted-matching (RTAM) for PP. Other options include applying identifiable characteristics or geometry to the fiducials. Lastly, after matching approximately six points (minimum 5.5), the PP matrix itself can be utilized to identify further matches.

A fully automatic matching for PP represents an important improvement in the workflow as it does not require precise projection knowledge between the 3D and the 2D points. It does require 3D knowledge of the fiducials and identification of the fiducials on 2D, but their precise correlation between 3D to 2D is not needed. Rather, a combinatorial optimization matching method (COMM) was implemented for this step to eliminate matching errors.

Mathematically, for a case comprising N (3D fiducials) and K (2D fiducials), where K <= N, the space of all possible combinations of K in N is:

$$N! / (K! * (N-K)!)\tag{65}$$

For each combination, there are K! permutations that must be searched to reach the best match. Therefore, given that unique combinations where order does need to match both 3D and 2D, the resulting numerical possibilities to investigate are:

$$N! / (N-K)!\tag{66}$$

As mentioned above, the solution of the PP transformation matrix derives from an overdetermined system that should be solved as a least squares error computation. Without additive information for each candidate match, the sum of the squares of the residuals of its solutions can be used as a metric of the matching quality. In principle, the automatic matching selection reduces to computation of all possible combinations and permutations keeping the one with the minimum least-squares error from its PP matrix solution. The previous crude approach produces very long computation times especially when the numbers K and N grow.

Singular value decomposition (SVD) is the most robust linear solution for PP matrix computation, but the suitability of QR-factorization, Gaussian Elimination, and Cholesky Factorization were explored. The methods were used in several test cases and each selected the same optimum match when minimizing mean square error (MSE). Cholesky factorization achieved convergence in less than a quarter of the time needed by SVD. Additional measures of the quality of the PP matrix can be utilized. For example, matrix condition number (CN) represents a ratio of the largest to smallest singular values (via SVD), which can eliminate singular or ill-conditioned systems. Theta, which should be 90 degrees (~1.57 radians), determines if the resultant vectors of the PP matrix are distorted as a measure of skew. Further, geometric constraints can be added if the estimated point source or detector plane are known. Generally, the fastest is MSE, which is not dependent on other factors or knowledge, but the addition of other thresholds ensures a consistent optimum result. Due to the nature of this approach and the prevalence of multicore CPUs/GPUs, a parallel implementation of the search process can also be implemented. For fiducials of n=8 and k=6, we get 20,160 possible combinations, and in combination with multiple thresholds, this represents a reasonable minimum for most CPUs.

RT fiducial tracking by moving focal spot location

As discussed above, the solution for RT is determined by the geometric properties of X-ray acquisition. If only some elements are known, a solution might be accomplished iteratively by changing the point source location and a tentative magnification multiplier until good overlap with fiducials is accomplished. If the geometry is largely unknown, but PP is possible, then PP can be used to compute the point source and image plane locations, which can subsequently be used in RT. This could be particularly useful when measurements are difficult to establish, but a relatively fixed setup is present.

**Figure 2:**
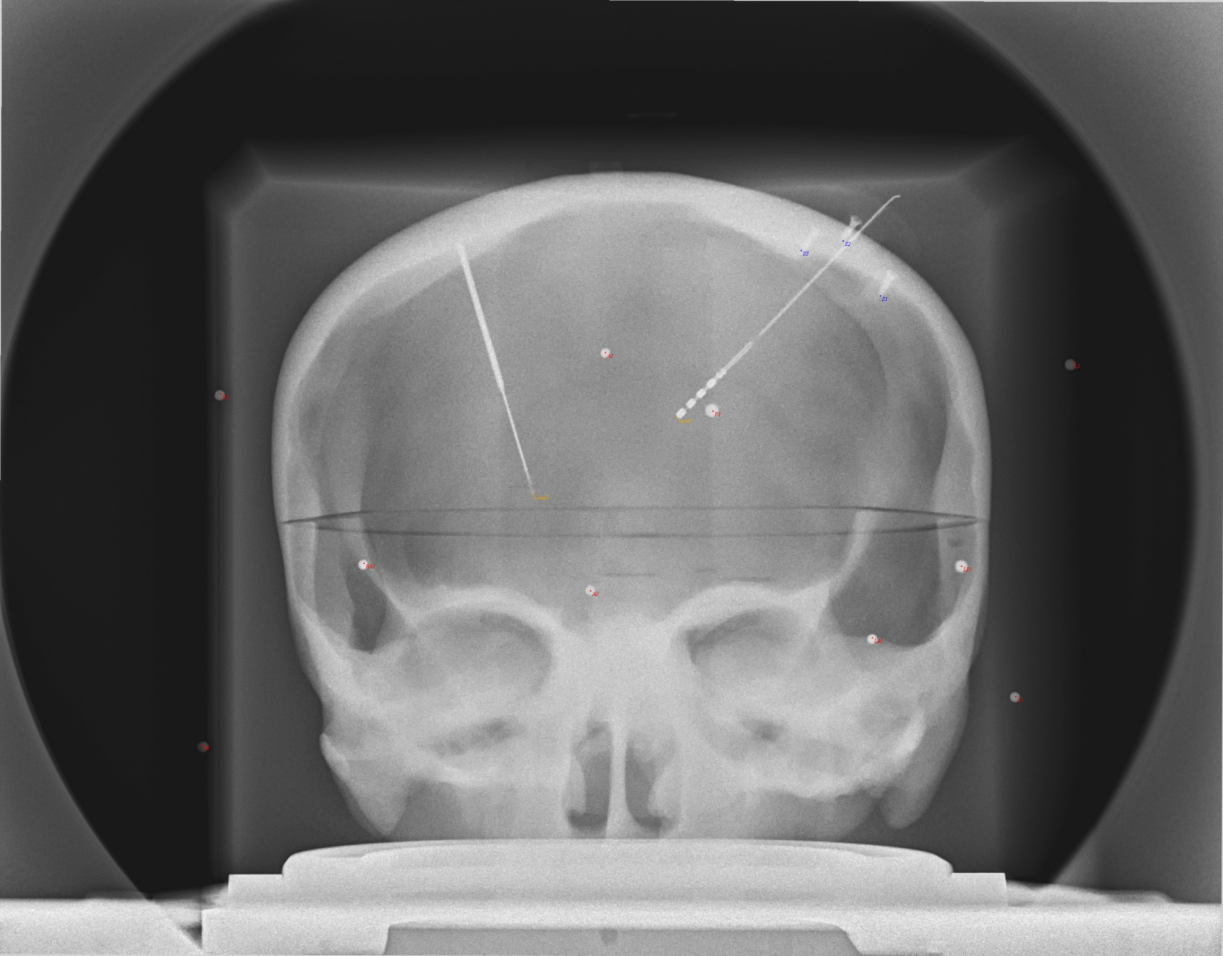
AP X-ray image of phantom prior to displacement of the focal spot. Note the good overlap of the projection points (red dots) with the objects (spheres, screw tips, electrode tips) on the image. AP: Antero-Posterior

**Figure 3:**
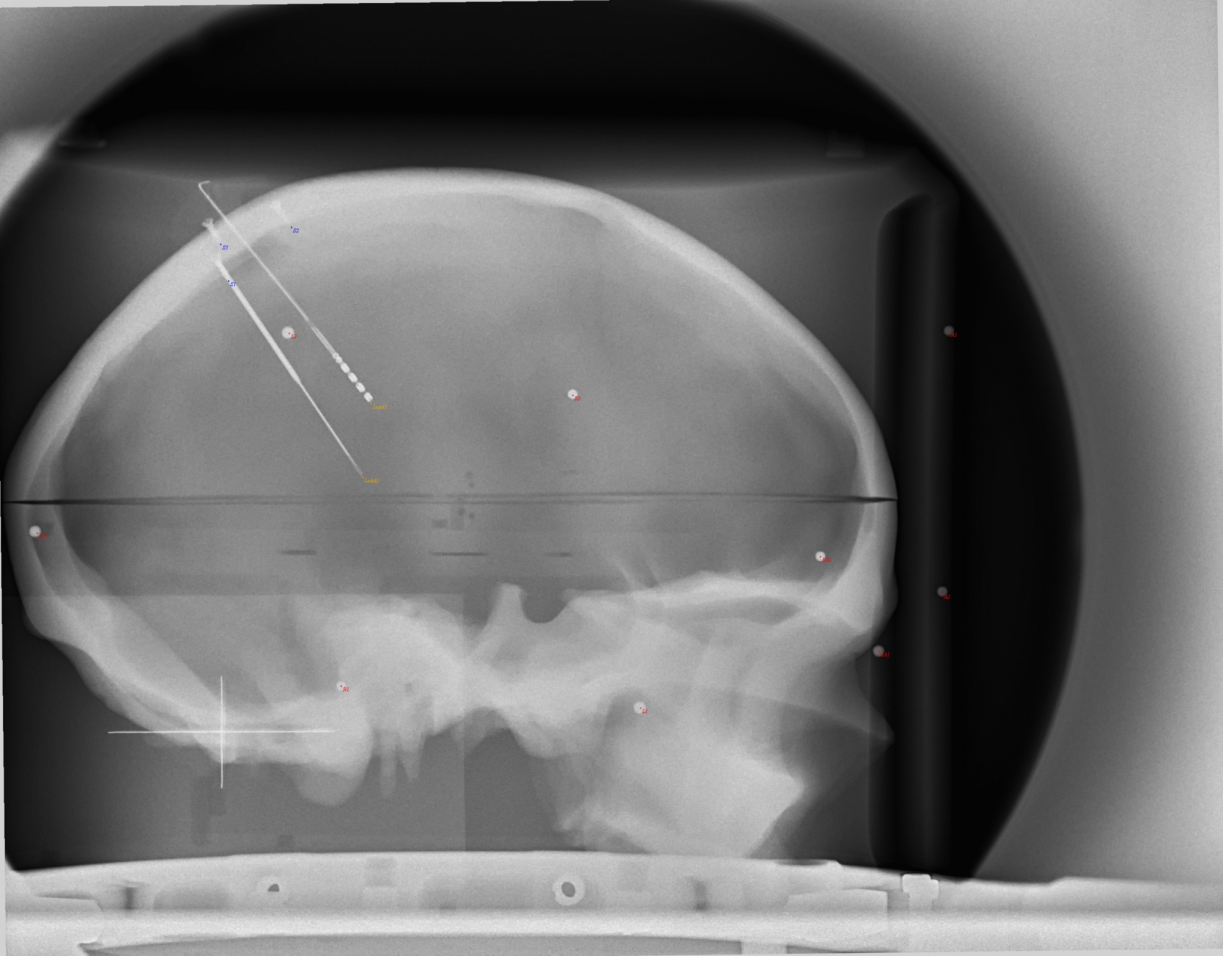
LAT X-ray image of phantom prior to displacement of the focal spot. Note the good overlap of the projection points (red dots) with the objects (spheres, screw tips, electrode tips) on the image. LAT: Lateral

**Figure 4:**
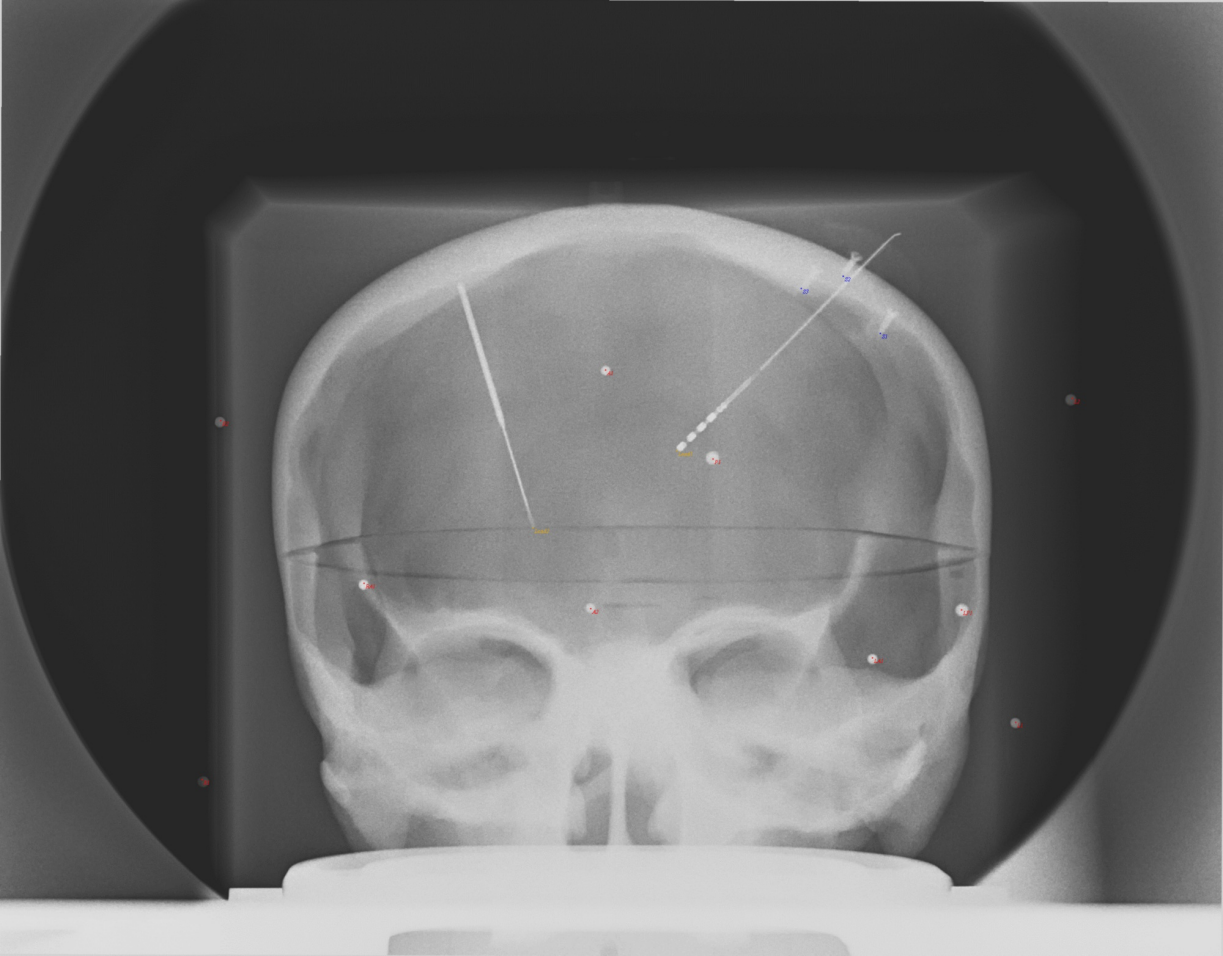
AP X-ray of phantom after the 20 mm displacement of the focal spot. Note the good overlap of the projection points (red dots) with the objects (spheres, screw tips, electrode tips) on the image that were slightly displaced from the previous image (Figure 2). This was accomplished by only changing the point source location, indicating that the fiducials on the display plane were predictable in this fixed setup. AP: Antero-Posterior; mm: millimeter

**Figure 5:**
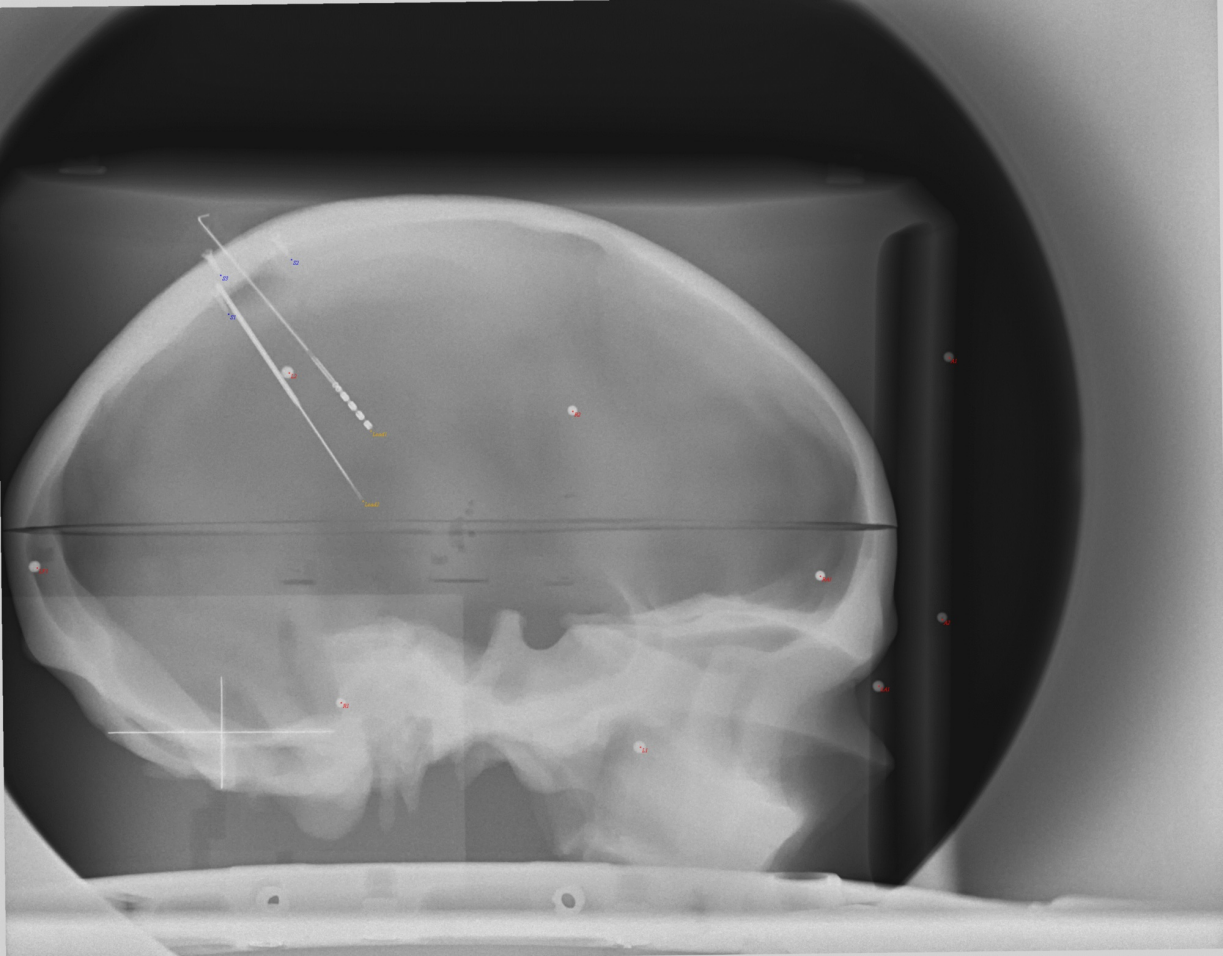
LAT X-ray of phantom after the 20 mm displacement of the focal spot. Note the good overlap of the projection points (red dots) with the objects (spheres, screw tips, electrode tips) on the image that were slightly displaced from the previous image (Figure 3). This was accomplished by only changing the point source location, indicating that the fiducials on the display plane were predictable in this fixed setup. LAT: Lateral; mm: millimeter

If the X-ray setup is fixed (Source, Object(s), and Detector), then known changes in geometry should be compensated quickly using RT. For example, we demonstrate a case of a fixed X-ray setup with a phantom skull and widely distributed geometric fiducials. In this example, PP is used to determine the origin point for RT in both AP and LAT views. Then the source position is displaced linearly by 20 mm and a new image is obtained. From PP, the prediction of the point source by Euclidean Error of the two exposures was 19.7 mm and 20.0 mm for AP and LAT, respectively. In RT, simple displacement of 20 mm produced points wherein the fiducials overlapped without added adjustments (Figures 2-5).

PP and orthogonal and non-orthogonal RT

The following tables demonstrate the mathematical solutions for RT and PP. Starting with orthogonal imaging (COA) in RT, point source and planar data can be combined with objects (fiducials) and line-detector plane intersections (Table 1). In this case, the source image distance (SID) is 1168 mm and the pixel spacing is 388 micrometer (um)/pixel when using 2D fluoroscopy mode using the O-arm2 (Medtronic, Dublin, Ireland). Computing the ray-plane intersections for all the relevant objects reveals the AP dimension to be constant at -515 (COA), which allows the use of the LAT and VERT coordinates to be translated directly to image (Table 2). Similarly, the LAT image has a constant LAT dimension of 513 (Table 3).

**Table 1:** 3D Point source and plane coordinates used for RT in 3D. SID is 1168 mm, which is known for O-arm2. Note the assumed orthogonality of the AP and LAT planes, which is not necessarily fixed. AP: Antero-Posterior; LAT: Lateral; VERT: Vertical; SID: Source image distance; RT: Ray tracing.

Point Source			Detector Plane		
	AP				
AP	653		-515	-515	-515
LAT	-7.5		0	100	0
VERT	93		0	0	100
	LAT				
AP	-5		0	100	0
LAT	-655		513	513	513
VERT	5		0	0	100

**Table 2:** Ray-to-detector plane intersections for actual data in the AP view using RT. Using knowledge of the point source and the detector plane locations in Table 1, combined with the object positions in AP, LAT, VERT in this table, the location of the intersections can be calculated. In addition, the angle of the intersection in degrees is shown relative to the normal of the AP plane. AP = Antero-Posterior; LAT = Lateral; VERT = Vertical; RT = Ray Tracing; AC = Anterior Commissure; PC = Posterior Commissure; Midline = Midline Structure on Falx; Target: Left STN = Target on Left Subthalamic Nucleus; 15mmAboveTarget: Left STN = 15 millimeters above target along stereotactic axis of left subthalamic nucleus; 75mmAboveTarget: Left STN = 75 millimeters above target along stereotactic axis of left subthalamic nucleus; RPPT = Right Posterior Pin Tip; RPPB = Right Posterior Pin Base; RPSS = Right Posterior Sphere Superior; RPSI = Right Posterior Sphere Inferior; RFPT = Right Frontal Pin Tip; RFPB = Right Frontal Pin Base; RFSS = Right Frontal Sphere Superior; RFSI = Right Frontal Sphere Inferior; LPPT = Left Posterior Pin Tip; LPPB = Left Posterior Pin Base; LPSS = Left Posterior Sphere Superior; LPSI = Left Posterior Sphere Inferior; LFSS = Left Frontal Sphere Superior; LFSI = Left Frontal Sphere Inferior; LFPT = Left Frontal Pin Tip; LFPB = Left Frontal Pin Base; Relecdelayed = Right Electrode Tip; Rscrew2 = Right Screw 2; RScrew1 = Right Screw 1.

Object				Intersection			
	AP	LAT	VERT	AP	LAT	VERT	90-angle of intersection
AC	8.7	-0.8	-11.2	-515	4.645895	-95.8959	9.205316
PC	-18.8	-0.4	-11.6	-515	4.84415	-88.8589	8.869997
Midline	5.7	-2.3	41.9	-515	1.882975	0.794222	4.53697
Target: Left STN	-8.65882	-12.4276	-15.7972	-515	-16.1984	-99.0553	9.347044
15mmAboveTarget: Left STN	-2.35342	-20.1082	-4.56108	-515	-29.9709	-80.8777	8.536699
75mmAboveTarget: Left STN	22.86817	-50.8308	40.38327	-515	-87.8171	-4.52933	6.17375
LFPB	89.4	-46.1	-5.6	-515	-87.4943	-111.338	10.64041
LFPT	74.6	-30	-4.1	-515	-52.9357	-103.08	9.777465
LFSI	77.8	-58	-34.2	-515	-110.045	-165.292	13.38359
LFSS	79.7	-54.5	-21.1	-515	-103.254	-139.459	12.1474
LPPB	-92.9	-59.6	-2.7	-515	-89.0831	-56.856	8.311116
LPPT	-75.5	-45.9	0.5	-515	-69.0665	-55.3047	7.827933
LPSI	-84	-65.3	-30.9	-515	-99.1016	-103.357	10.50932
LPSS	-85	-64.2	-19.6	-515	-97.2366	-85.207	9.694069
RFPB	87.8	49	-4.5	-515	109.2587	-108.486	11.27558
RFPT	72.5	33.5	-2.9	-515	74.9944	-99.9564	10.1855
RFSI	77.1	60.6	-38.4	-515	130.6156	-173.496	14.41237
RFSS	79.3	56.7	-19.7	-515	123.2052	-136.447	12.73938
RPPB	-94.1	48.5	-3.9	-515	80.04919	-58.4914	8.51973
RPPT	-77	34.1	-1	-515	59.06	-57.4	8.015313
RPSI	-86.6	58.3	-26.2	-515	96.41347	-95.2445	10.43098
RPSS	-87.4	57.5	-21.5	-515	95.03917	-87.6267	10.08335
Relecdelayed	-8.7	12.3	-16	-515	27.44998	-99.4014	9.504477
Rscrew2	42.1	40	41.4	-515	83.31683	-5.65575	6.549156
RScrew1	19.6	46.3	46.3	-515	91.70808	6.884433	6.417344

**Table 3:** Ray-to-detector plane intersections for actual data in LAT view using RT. Using knowledge of the point source and the detector plane locations in Table 1, combined with the object positions in AP, LAT, VERT in this table, the location of the intersections can be calculated. In addition, the angle of the intersection in degrees is shown relative to the normal of the LAT plane. AP = Antero-Posterior; LAT = Lateral; VERT = Vertical; RT = Ray Tracing; AC = Anterior Commissure; PC = Posterior Commissure; Midline = Midline structure on Falx; Target: Left STN = Target on Left Subthalamic Nucleus; 15mmAboveTarget: Left STN = 15 millimeters above target along stereotactic axis of left subthalamic nucleus; 75mmAboveTarget: Left STN = 75 millimeters above target along stereotactic axis of left subthalamic nucleus; RPPT = Right Posterior Pin Tip; RPPB = Right POsterior Pin Base; RPSS = Right Posterior Sphere Superior; RPSI = Right Posterior Sphere Inferior; RFPT = Right Frontal Pin Tip; RFPB = Right Frontal Pin Base; RFSS = Right Frontal Sphere Superior; RFSI = Right Frontal Sphere Inferior; LPPT = Left Posterior Pin Tip; LPPB = Left Posterior Pin Base; LPSS = Left Posterior Sphere Superior; LPSI = Left Posterior Sphere Inferior; LFSS = Left Frontal Sphere Superior; LFSI = Left Frontal Sphere Inferior; LFPT = Left Frontal Pin Tip; LFPB = Left Frontal Pin Base; Relecdelayed = Right Electrode Tip; Rscrew2 = Right Screw 2; RScrew1 = Right Screw 1.

Object				Intersection			
	AP	LAT	VERT	AP	LAT	VERT	90-angle of intersection
AC	8.7	-0.8	-11.2	19.4598	513	-23.9233	1.8575
PC	-18.8	-0.4	-11.6	-29.6233	513	-24.6193	1.888784
Midline	5.7	-2.3	41.9	14.14754	513	71.03217	3.368729
Target: Left STN	-8.65882	-12.4276	-15.7972	-11.6506	513	-32.8029	1.882207
15mmAboveTarget: Left STN	-2.35342	-20.1082	-4.56108	-0.13113	513	-12.5894	0.895213
75mmAboveTarget: Left STN	22.86817	-50.8308	40.38327	48.87568	513	73.40412	4.263447
LFPB	89.4	-46.1	-5.6	176.0793	513	-15.3331	8.867123
LFPT	74.6	-30	-4.1	143.7565	513	-12.0061	7.304882
LFSI	77.8	-58	-34.2	156.994	513	-71.6928	8.724066
LFSS	79.7	-54.5	-21.1	159.7454	513	-45.7657	8.395892
LPPB	-92.9	-59.6	-2.7	-177.434	513	-10.1051	8.429719
LPPT	-75.5	-45.9	0.5	-140.19	513	-3.62912	6.615612
LPSI	-84	-65.3	-30.9	-161.473	513	-66.106	8.371011
LPSS	-85	-64.2	-19.6	-163.158	513	-43.6337	8.063262
RFPB	87.8	49	-4.5	148.9636	513	-10.7614	7.548125
RFPT	72.5	33.5	-2.9	126.4742	513	-8.40189	6.455383
RFSI	77.1	60.6	-38.4	129.0034	513	-65.8373	7.394104
RFSS	79.3	56.7	-19.7	133.3482	513	-35.5362	7.036344
RPPB	-94.1	48.5	-3.9	-152.93	513	-9.7764	7.253758
RPPT	-77	34.1	-1	-127.037	513	-5.16979	5.985381
RPSI	-86.6	58.3	-26.2	-138.617	513	-46.0887	6.982518
RPSS	-87.4	57.5	-21.5	-140.078	513	-38.4414	6.926503
Relecdelayed	-8.7	12.3	-16	-11.4762	513	-31.7571	1.830254
Rscrew2	42.1	40	41.4	74.15511	513	66.17295	4.895392
RScrew1	19.6	46.3	46.3	35.97077	513	73.78426	3.921262

**Figure 6:**
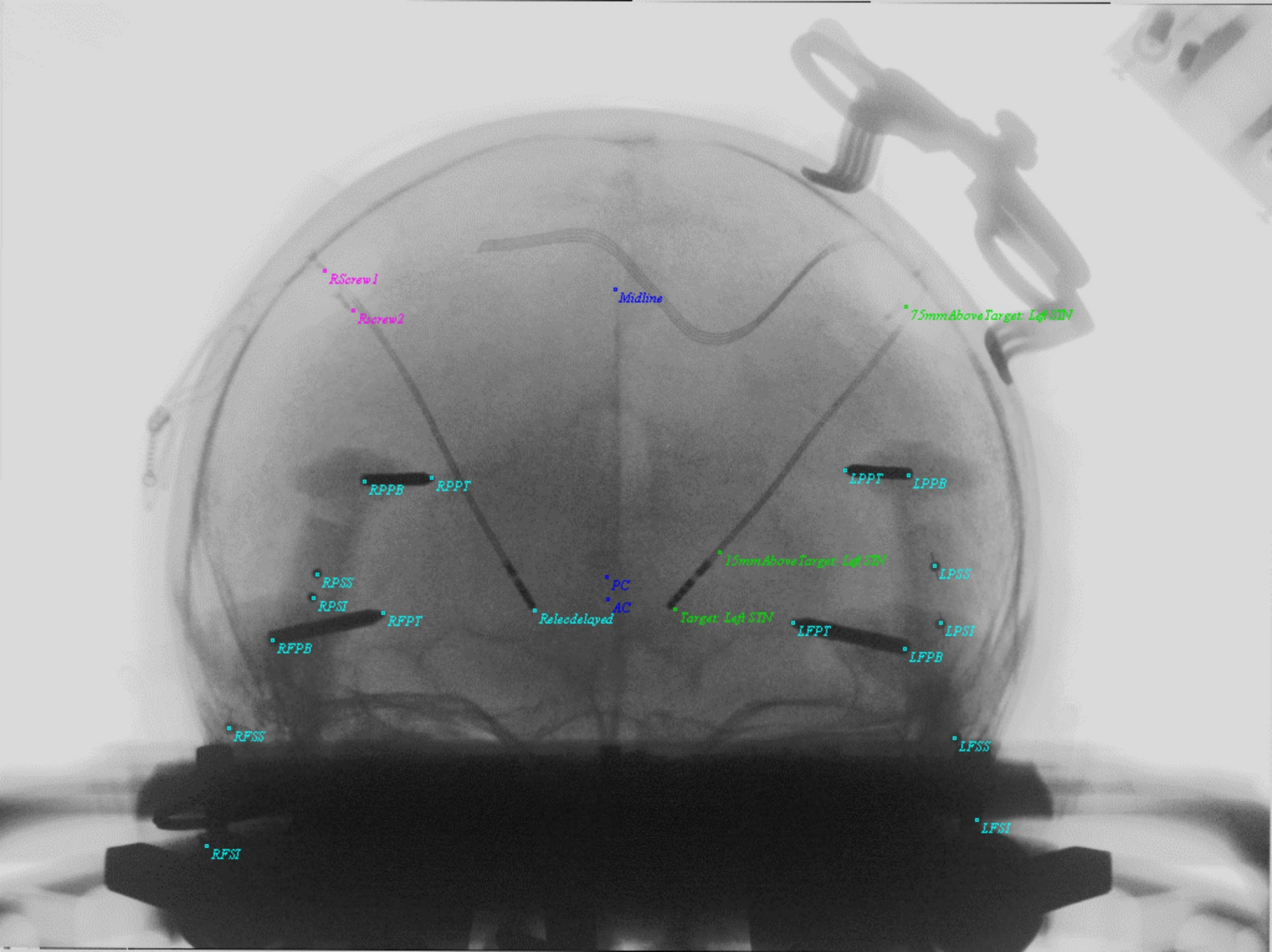
AP Image demonstrating the applied PP AP matrix to objects and overlap in images. Taking formula's 10-12, U (horizontal) and V (vertical) display coordinates are computed for all 3D objects and introduced into the display. Cyan colors show pin tips, pin bases, and the right DBS electrode. Screw tips from the DBS retaining cap were also included here. Anterior commissure (AC), Posterior commissure (PC), and Midline are demonstrated in blue. The left DBS electrode and original trajectory from left STN target, 15 mm above target and 75 mm above target are in green. DBS = Deep Brain Stimulation; AP = Antero-Posterior; PP = Perspective Projection; AC = Anterior Commissure; PC = Posterior Commissure; Midline = Midline structure on Falx; Target: Left STN = Target on Left Subthalamic Nucleus; 15mmAboveTarget: Left STN = 15 millimeters above target along stereotactic axis of left subthalamic nucleus; 75mmAboveTarget: Left STN = 75 millimeters above target along stereotactic axis of left subthalamic nucleus; RPPT = Right Posterior Pin Tip; RPPB = Right POsterior Pin Base; RPSS = Right Posterior Sphere Superior; RPSI = Right Posterior Sphere Inferior; RFPT = Right Frontal Pin Tip; RFPB = Right Frontal Pin Base; RFSS = Right Frontal Sphere Superior; RFSI = Right Frontal Sphere Inferior; LPPT = Left Posterior Pin Tip; LPPB = Left Posterior Pin Base; LPSS = Left Posterior Sphere Superior; LPSI = Left Posterior Sphere Inferior; LFSS = Left Frontal Sphere Superior; LFSI = Left Frontal Sphere Inferior; LFPT = Left Frontal Pin Tip; LFPB = Left Frontal Pin Base; Relecdelayed = Right Electrode Tip; Rscrew2 = Right Screw 2; RScrew1 = Right Screw 1.

**Figure 7:**
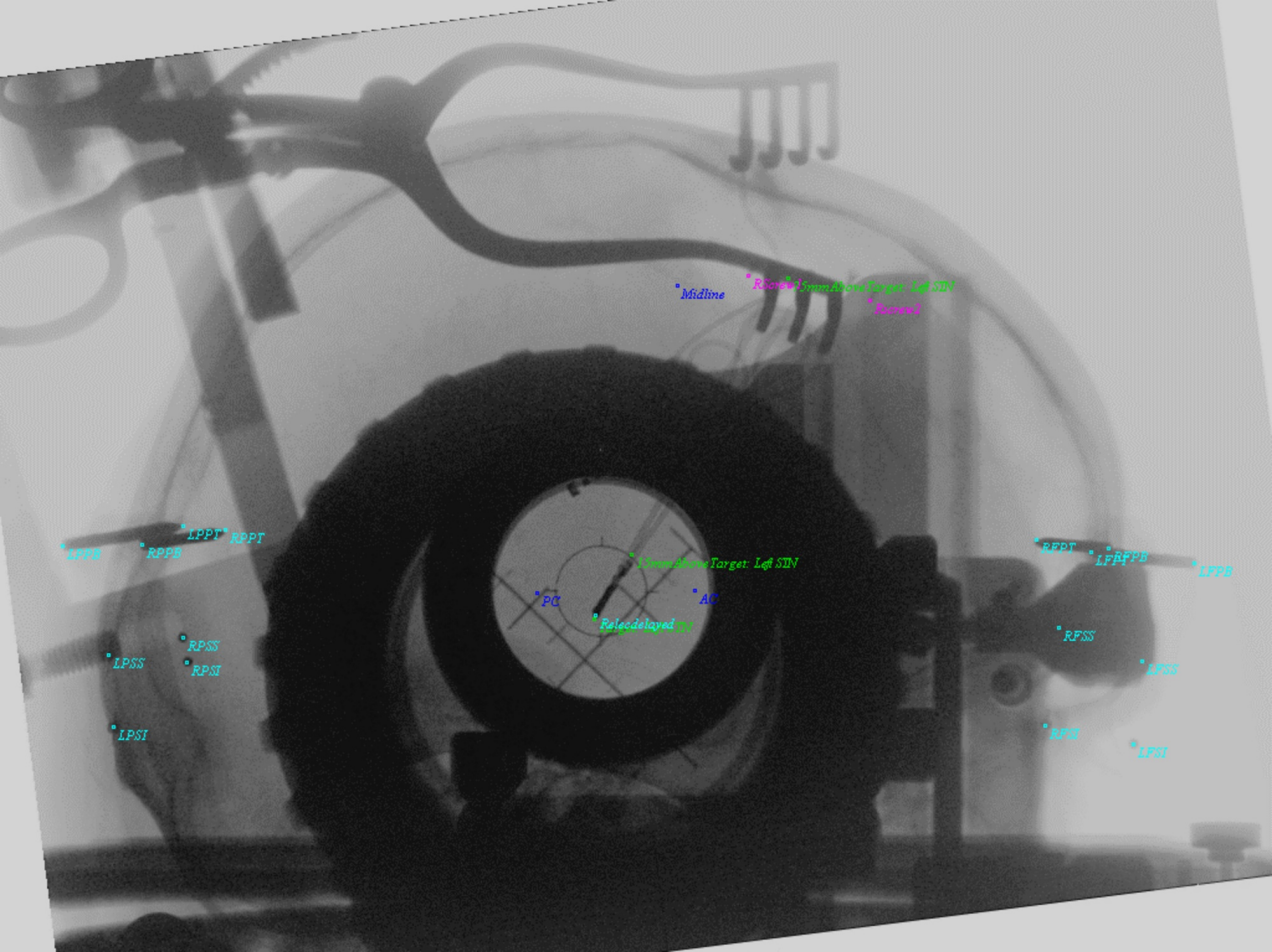
LAT Image demonstrating the applied PP LAT matrix to objects and overlap in images. Taking formula's 10-12, U (horizontal) and V (vertical) display coordinates are computed for all 3D objects and introduced into the display. Cyan colors show pin tips, pin bases, and the right DBS electrode. Screw tips from the DBS retaining cap were also included here. Anterior commissure (AC), Posterior commissure (PC), and Midline are demonstrated in blue. The left DBS electrode and original trajectory from left STN target, 15 mm above target and 75 mm above target are in green. DBS = Deep Brain Stimulation; LAT = Lateral; PP = Perspective Projection; AC = Anterior Commissure; PC = Posterior Commissure; Midline = Midline structure on Falx; Target: Left STN = Target on Left Subthalamic Nucleus; 15mmAboveTarget: Left STN = 15 millimeters above target along stereotactic axis of left subthalamic nucleus; 75mmAboveTarget: Left STN = 75 millimeters above target along stereotactic axis of left subthalamic nucleus; RPPT = Right Posterior Pin Tip; RPPB = Right POsterior Pin Base; RPSS = Right Posterior Sphere Superior; RPSI = Right Posterior Sphere Inferior; RFPT = Right Frontal Pin Tip; RFPB = Right Frontal Pin Base; RFSS = Right Frontal Sphere Superior; RFSI = Right Frontal Sphere Inferior; LPPT = Left Posterior Pin Tip; LPPB = Left Posterior Pin Base; LPSS = Left Posterior Sphere Superior; LPSI = Left Posterior Sphere Inferior; LFSS = Left Frontal Sphere Superior; LFSI = Left Frontal Sphere Inferior; LFPT = Left Frontal Pin Tip; LFPB = Left Frontal Pin Base; Relecdelayed = Right Electrode Tip; Rscrew2 = Right Screw 2; RScrew1 = Right Screw 1.

The following tables (Tables 4-8) demonstrate the PP solution using 3D coordinates combined with scaled (in millimeters) U and V screen coordinates for analysis in both AP and LAT views. The complete PP matrices are computed using SVD with 22 and 26 equations in AP and LAT, respectively (Table 6). Applying the matrices to the images for all the hyperdense objects (fiducials, pins, electrode tips) shows overlap in the AP and LAT image (Figures 6, 7). The next step is to use PP to develop the geometry and compare the results to RT (Table 7). Finally, we apply biplanar imaging to localize the 3D position (STiL) in both RT and PP. The solution in RT can be derived from a line-line intersection problem using parametric equations for the lines to solve for the parameters r, which describes the closest point of intersection (equations 67-69). After r is solved, the values for x, y, and z can be readily calculated by plugging the value back into each parametric equation. A skew ray (line-line) intersection computation generating the closest points of intersection on each ray allows for a Euclidean distance between those points serving as a quality check between the two images. The biplanar point solution for PP can be derived by first generating complete matrices in AP and LAT (equations 70, 71). Then the U and V screen coordinates are used in the four planar systems of equations to solve x, y, and z simultaneously (equations 72-75). The results for the left electrode tip, converted to MidACPC coordinates demonstrate a 3D Euclidean error of 0.48 mm and 0.30 mm relative to immediate postoperative CT for RT and PP, respectively (Table 8).

**Table 4:** AP data used for input into PP computation. Here, there is a combination of 3D object points (AP, LAT, VERT) and 2D points on the display plane (U, V). AP = Antero-Posterior; LAT = Lateral; VERT = Vertical; U = Horizontal Axis of Display Plane; V = Vertical Axis of Display Plane; PP = Perspective Projection; RPPT = Right Posterior Pin Tip; RPSS = Right Posterior Sphere Superior; RPSI = Right Posterior Sphere Inferior; RFPT = Right Frontal Pin Tip; RFSS = Right Frontal Sphere Superior; LPPT = Left Posterior Pin Tip; LPSS = Left Posterior Sphere Superior; LPSI = Left Posterior Sphere Inferior; LFSS = Left Frontal Sphere Superior; LFPT = Left Frontal Pin Tip; RFPB = Right Frontal Pin Base.

	Object	AP	LAT	VERT	U	V
1	RPPT	-77	34.1	-1	134.947	149.564
2	RPSS	-87.4	57.5	-21.5	98.86	179.35
3	RPSI	-86.6	58.3	-26.2	97.348	186.75
4	RFPT	72.5	33.5	-2.9	119.836	191.75
5	RFSS	79.3	56.7	-19.7	71.647	227.815
6	LPPT	-75.5	-45.9	0.5	263.74	146.983
7	LPSS	-85	-64.2	-19.6	291.933	177.068
8	LPSI	-84	-65.3	-30.9	293.507	195.25
9	LFSS	79.7	-54.5	-21.1	298.518	230.707
10	LFPT	74.6	-30	-4.1	247.177	194.656
11	RFPB	87.8	49	-4.5	85.07	199.64

**Table 5:** LAT data used for input into PP computation. Here, there is a combination of 3D object points (AP, LAT, VERT) and 2D points on the display plane (U, V). AP = Antero-Posterior; LAT = Lateral; VERT = Vertical; U = Horizontal Axis of Display Plane; V = Vertical Axis of Display Plane; PP = Perspective Projection; LPPT = Left Posterior Pin Tip; RPPT = Right Posterior Pin Tip; RPSS = Right Posterior Sphere Superior; RPSI = Right Posterior Sphere Inferior; LPSI = Left Posterior Sphere Inferior; RFSI = Right Frontal Sphere Inferior; LFSI = Left Frontal Sphere Inferior; RFPT = Right Frontal Pin Tip; LFPB = Left Frontal Pin Base; RPPB = Right Posterior Pin Base; LPPB = Left Posterior Pin Base; LPSS = Left Posterior Sphere Superior; RScrew1 = Right Screw 1.

	Object	AP	LAT	VERT	U	V
1	LPPT	-75.5	-45.9	0.5	56.622	164.432
2	RPPT	-77	34.1	-1	69.602	166.417
3	RPSS	-87.4	57.5	-21.5	56.85	199.482
4	RPSI	-86.6	58.3	-26.2	58.369	206.983
5	LPSI	-84	-65.3	-30.9	35.246	227.713
6	RFSI	77.1	60.6	-38.4	326.496	226.484
7	LFSI	77.8	-58	-34.2	353.981	232.634
8	RFPT	72.5	33.5	-2.9	323.107	169.639
9	LFPB	89.4	-46.1	-5.6	372.72	175.96
10	RPPB	-94.1	48.5	-3.9	44.513	170.195
11	LPPB	-92.9	-59.6	-2.7	20.165	170.449
12	LPSS	-85	-64.2	-19.6	33.29	204.795
13	RScrew1	19.6	46.3	46.3	233.346	86.749

**Table 6:** AP and LAT Matrices resulting from SVD calculation in PP from data in Tables 4, 5. For AP Matrix: MSE 0.312 mm; Matrix Condition Number: 55014.68. For LAT Matrix: MSE 0.432 mm; Matrix Condition Number: 78991.78. These data are displayed as a 3x4 format (see equation 22). AP = Antero-Posterior; LAT = Lateral; SVD = Singular Value Decomposition; MSE = Mean Square Error.

	PP Matrices		
AP Matrix (3x4)			
-0.30417	-1.7812	0.003801	188.3312
0.001436	0.012411	-1.78216	165.1482
-0.00152	6.84E-05	-2.21E-05	1
LAT Matrix (3x4)			
1.785236	0.29387	0.015113	201.1355
0.003202	0.236093	-1.77785	164.6433
2.13E-05	0.001521	7.00E-05	1

**Table 7:** Solutions computed from PP Matrices derived from Table 6 reveal the geometry of the biplanar imaging. Here we are able to compute the point source in AP and LAT. In addition, K-matrix (intrinsic matrix) components are shown including principal point, skew angle (theta), and a simple average between alpha and beta. The planes are computed using the focal distance obtained from a quadratic solution and the normal of the detector plane. The normal of the computed detector plane relative to the normal of the normalized plane is also shown as the "AP/LAT Normal to Normal Angle." AP = Antero-Posterior; LAT = Lateral; VERT = Vertical; PP = Perspective Projection; Combined = Simple average of Alpha and Beta for focal length calculation; Alpha = Alpha length from intrinsic matrix for focal length calculation; Beta = Beta length from intrinsic matrix for focal length calculation; $U_{0} \: and \: V_{0} \: = \:  principal \: point$ Theta = Angle used to determine skew angle (here presented in radians); Distance from Point Source to Plane = Focal Length, which was calculated using Quadratic Solution; AP/LAT Normal-to-normal Angle (degrees) = normal of computed detector plane relative to the associated normal of a normalized plane.

Solutions in AP and LAT Computed from PP					
AP Point Source (AP, LAT, VERT):					
655.7883	-6.05706	93.15404			
Combined	Alpha	Beta	Uo	Vo	Theta
1173.785	1177.416	1170.154	146.9495	16.4229	1.581723
AP Plane Calculation using Intrinsic Matrix from PP (AP, LAT, VERT):					
-511.98	0	0			
-513.434	0	100			
-516.476	-100	0			
Focal Distance AP (Quadratic Solution):					
1168.091					
AP Normal-to-Normal Angle (degrees): 2.70572703691867					
LAT Point Source (AP, LAT, VERT):					
-4.4651	-657.583	5.275153			
Combined	Alpha	Beta	Uo	Vo	Theta
1171.379	1169.453	1173.304	209.5675	101.2284	1.571102
LAT Plane Calculation using Intrinsic Matrix from PP Reverse Cross Product (AP, LAT, VERT):					
0	515.5404	0			
0	510.9394	100			
-100	516.9381	0			
Focal Distance LAT (Quadratic Solution):					
1171.589					
LAT Normal-to-Normal Angle (degrees): 2.75303244159637					

**Table 8:** Comparison of target localization coordinates for an electrode tip in a single case. Here, MidACPC coordinates are presented. All Euclidean distances are compared to the "gold standard" postoperative CT, including the final intraoperative image (O-arm2) and the originally planned target. RT and PP fair well relative to the post-op CT with Euclidean distances of 0.48 mm and 0.30 mm as compared to O-arm2 of 0.41 mm. AP = Antero-Posterior; LAT = Lateral; VERT = Vertical; MidACPC = Middle of the Anterior Commissure to Posterior Commissure point; RT = Ray Tracing; PP = Perspective Projection; Rel Post-op CT = Euclidian Distance relative to the post-op CT; Rel to O-arm2 = Euclidian Distance relative to the intraoperative O-arm2; Rel to Plan Target = Euclidian Distance relative to original planned target.

	Planned Target	O-arm2	CT	RT	PP
AP	-3.5	-3.02	-3.16	-3.32	-3.43
LAT	-12	-11.15	-10.8	-10.6	-10.74
VERT	-4	-3.71	-3.88	-3.47	-3.76
Rel Post-op CT	1.252996	0.413521	0	0.483425	0.301496
Rel to O-arm2	1.018332	0	0.413521	0.670895	0.581979
Rel to Plan Target	0	1.018332	1.252996	1.507747	1.284562

$$x_{AP} + a_{AP}*r_{AP} = x_{LAT} + a_{LAT}*r_{LAT}\tag{67}$$

$$y_{AP} + b_{AP}*r_{AP} = y_{LAT} + b_{LAT}*r_{LAT}\tag{68}$$

$$z_{AP} + c_{AP}*r_{AP} = z_{LAT} + c_{LAT}*r_{LAT}\tag{69}$$

$$\begin{bmatrix} AP_{11} & AP_{12} & AP_{13} \\ AP_{21} & AP_{22} & AP_{23}\\ AP_{31} & AP_{32} & AP_{33}\\ AP_{41} & AP_{42} & AP_{43} \end{bmatrix}\tag{70}$$

$$\begin{bmatrix} LAT_{11} & LAT_{12} & LAT_{13} \\ LAT_{21} & LAT_{22} & LAT_{23}\\ LAT_{31} & LAT_{32} & LAT_{33}\\ LAT_{41} & LAT_{42} & LAT_{43} \end{bmatrix}\tag{71}$$

$$x(AP_{11}-AP_{13}U_{AP}) + y(AP_{21}-AP_{23}U_{AP}) + z(AP_{31}-AP_{33}U_{AP}) - U_{AP}AP_{43} + AP_{41} = 0 \tag{72}$$

$$x(AP_{12}-AP_{13}V_{AP}) + y(AP_{22}-AP_{23}V_{AP}) + z(AP_{32}-AP_{33}V_{AP}) - V_{AP}AP_{43} + AP_{42} = 0 \tag{73}$$

$$x(LAT_{11}-LAT_{13}U_{LAT}) + y(LAT_{21}-LAT_{23}U_{LAT}) + z(LAT_{31}-LAT_{33}U_{LAT}) - U_{LAT}LAT_{43} + LAT_{41} = 0 \tag{74}$$

$$x(LAT_{12}-LAT_{13}V_{LAT}) + y(LAT_{22}-LAT_{23}V_{LAT}) + z(LAT_{32}-LAT_{33}V_{LAT}) - V_{LAT}LAT_{43} + LAT_{42} = 0 \tag{75}$$

**Figure 8:**
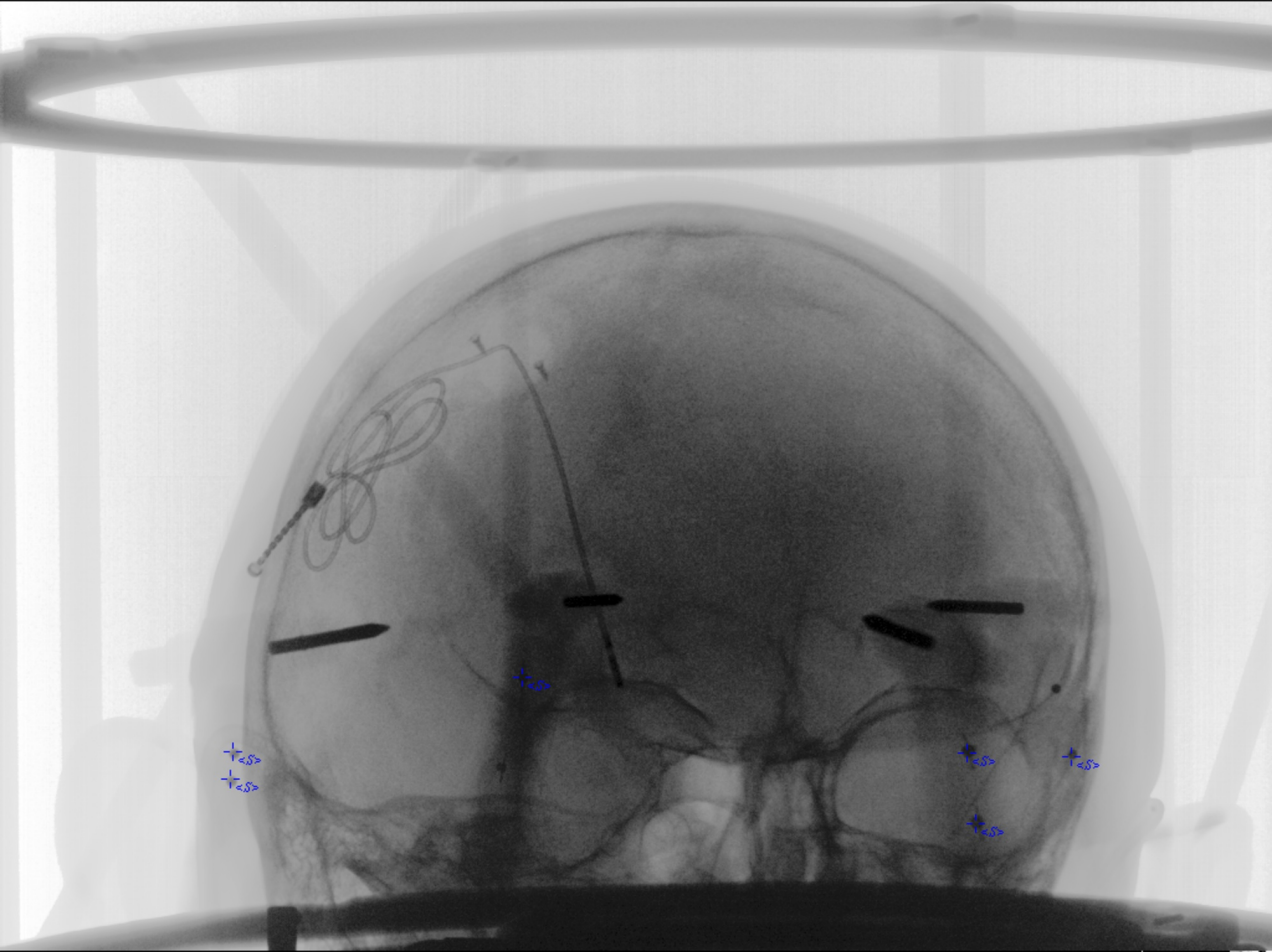
Oblique image with a setting of combinatorial points as unknowns 'S'. These screen positions are the input data for which the association between 3D fiducials and 2D image points will be generated in PP. PP = Perspective Projection

**Figure 9:**
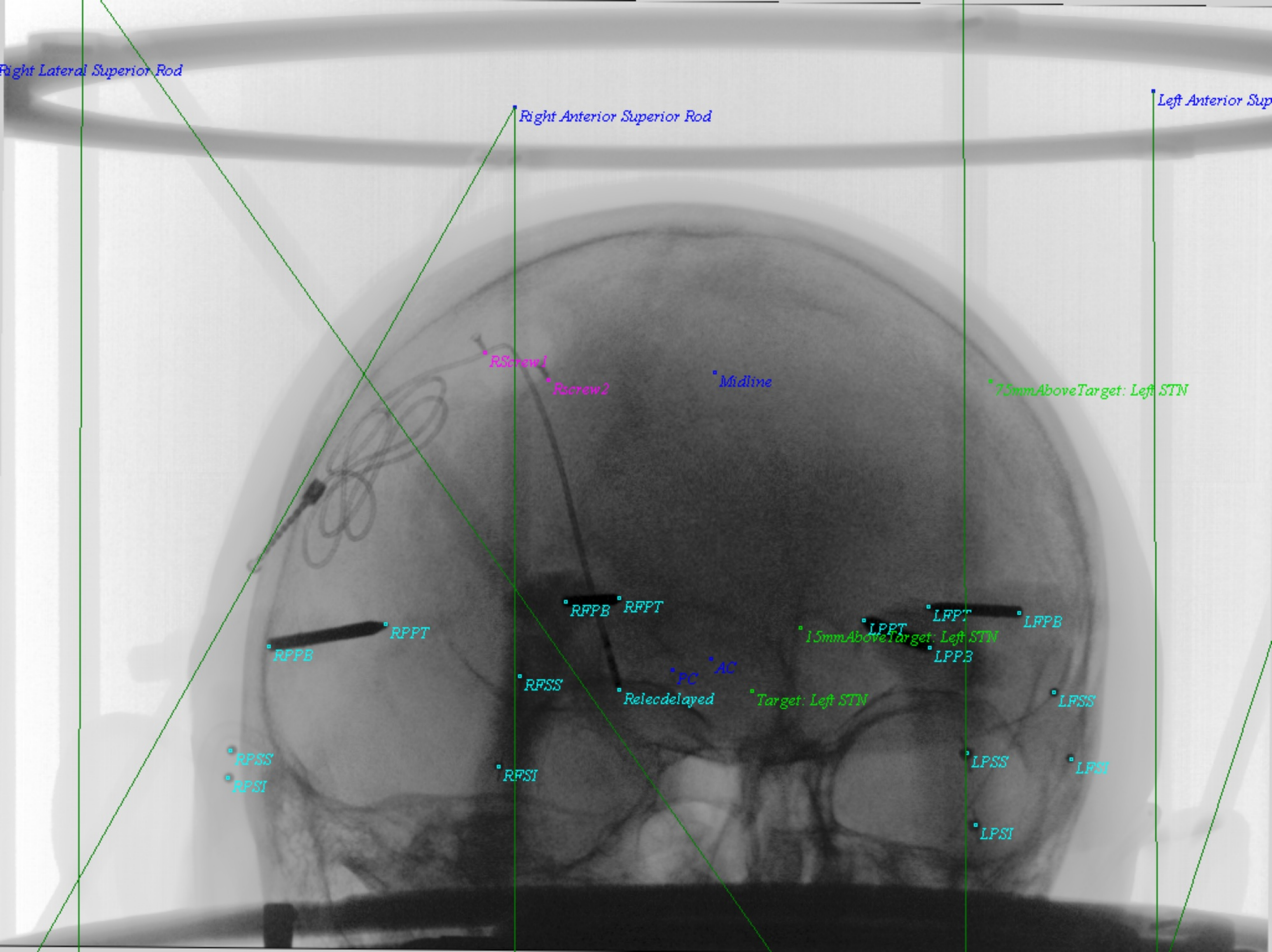
Oblique projection from RT. Input data started with PP matrix, the geometry was computed for point source and image plane, and then placed into RT for plane intersection and normalization. The RT projection shows excellent overlap of objects and N-localizer bars (green) of BRWLF (reference 3). Cyan colors show pin tips, pin bases, and the right DBS electrode. Screw tips from the DBS retaining cap were also included here. Anterior commissure (AC), Posterior commissure (PC), and Midline are demonstrated in blue. The left DBS electrode and original trajectory from the left STN target, 15 mm above the target, and 75 mm above the target are in green. In addition, the N-localizer bars emerge from points above the ring (blue). AC = Anterior Commissure; PC = Posterior Commissure; Midline = Midline structure on Falx; Target: Left STN = Target on Left Subthalamic Nucleus; 15mmAboveTarget: Left STN = 15 millimeters above target along stereotactic axis of left subthalamic nucleus; 75mmAboveTarget: Left STN = 75 millimeters above target along stereotactic axis of left subthalamic nucleus; RPPT = Right Posterior Pin Tip; RPPB = Right Posterior Pin Base; RPSS = Right Posterior Sphere Superior; RPSI = Right Posterior Sphere Inferior; RFPT = Right Frontal Pin Tip; RFPB = Right Frontal Pin Base; RFSS = Right Frontal Sphere Superior; RFSI = Right Frontal Sphere Inferior; LPPT = Left Posterior Pin Tip; LPPB = Left Posterior Pin Base; LPSS = Left Posterior Sphere Superior; LPSI = Left Posterior Sphere Inferior; LFSS = Left Frontal Sphere Superior; LFSI = Left Frontal Sphere Inferior; LFPT = Left Frontal Pin Tip; LFPB = Left Frontal Pin Base; Relecdelayed = Right Electrode Tip; Rscrew2 = Right Screw 2; RScrew1 = Right Screw 1; BRWLF = Brown-Roberts-Wells Localizer Frame.

Next, we consider the situation of uncertain correlation between 3D points to 2D points. For this, the same patient case discussed above now includes an initial oblique image with the N-localizer positioned (Figure 8). We start the solution by using a combinatorial approach. Various thresholds can be analyzed, but here we assume no specific knowledge and use MSE and the matrix condition number (CN), but include the theta angle result (Table 9). The combination of low MSE, low CN, and theta closest to 1.57 radians identify the result. The next step is to use the geometric analysis of the point source and plane location (Table 10). These data are then placed in RT, but require normalization along the AP axis to make COA (Table 11). The final result produced by RT shows excellent overlap (Figure 9). Note that the projection can also be performed using PP. Because both methods project the scalar distance on the detector plane, data from RT and PP should be similar. Here, we randomly chose the AC point to Midline point yielding a distance in RT of 89.604 mm to PP of 89.694 mm. Finally, in RT, the result between detector plane intersection and display plane (normalized) is exactly the same (89.604 mm).

**Table 9:** Combinatorial approach in the image reveals 12 possible combinations. Two Thresholds (MSE <0.05 and Matrix Condition Number <300000) were implemented but the third data point (theta) is presented. Match 7 is the correct combination here for which theta (in radians) is also closest to pi/2 (~1.57). Combinatorials = the combinatorial match which accommodated the thresholds; MSE = Mean Square Error; Condition = Matrix Condition or Matrix Number; Theta = Angle used to determine skew angle (here presented in radians).

Combinatorials	MSE	Condition	Theta (radians)
1	0.0214	234536	0.7775
2	0.0294	272250	1.9139
3	0.0005	254621	1.304
4	0.0353	208276	1.1685
5	0.0193	278288	3.0104
6	0.0337	205428	1.1826
7	0.0141	294228	1.5808
8	0.0283	271562	0.0184
9	0.0386	285403	0.9659
10	0.0219	238902	0.8718
11	0.0498	241089	0.2657
12	0.0438	244903	1.2075

**Table 10:** PP Matrix Result (as displayed in 3x4 form) and Geometric Analysis. Here we are able to compute the point source in the AP oblique. In addition, K-matrix (intrinsic matrix) components are shown including principal point, skew angle, and a simple average between alpha and beta. The plane is computed using the focal distance obtained from a quadratic solution and the normal of the detector plane. The normal of the computed detector plane relative to the normalized plane is also shown (Normal-to-Normal Angle). Note that the expected focal distance for O-arm2 is 1168 mm, whereas here we compute 1161.68 mm. AP = Antero-Posterior; LAT = Lateral; VERT = Vertical; PP = Perspective Projection; Combined = Simple average of Alpha and Beta for focal length calculation; Alpha = alpha length from intrinsic matrix for focal length calculation; Beta = beta length from intrinsic matrix for focal length calculation $U_{0} \: and \: V_{0} \: =  \: principal \: point$ Theta = Angle used to determine skew angle (here presented in radians) Distance from Point Source to Plane = Focal Length, which was calculated using Quadratic Solution Normal-to-Normal Angle in degrees = detector plane normal compared to the normalized plane

PP Result					
AP Matrix (3x4)					
0.706033	-1.57036	0.001052	217.2948		
0.192262	0.05844	-1.70128	187.6501		
0.001416	0.000382	4.55E-05	1		
AP Oblique Point Source (AP, LAT, VERT):					
-663.821	-160.061	29.78255			
Combined	Alpha	Beta	Uo	Vo	Theta
1160.582	1158.306	1162.858	185.9213	100.8434	1.570177
Plane Calculation using Intrinsic Matrix from PP Reverse Cross Product (AP, LAT, VERT):					
497.7189	0	0			
494.5064	0	100			
524.6757	-100	0			
Focal Distance (Quadratic Solution):					
1161.684					
Normal-to-Normal Angle (degrees): 15.1883448297578					

**Table 11:** RT Projection from geometric analysis of PP (Table 10) along with normalization of the axis to keep AP intersection constant. Here we take the point source and plane data from Table 10, computed from PP, and then generate detector plane intersection. Because the detector plane is off-axis (not COA), a normalization procedure is computed as the corrected form, which forces all AP distances to a constant value (COA). Then the LAT/VERT of the corrected can be translated/rotated and positioned over the image. AC = Anterior Commissure; PC = Posterior Commissure; Midline = Midline structure on Falx; Target: Left STN = Target on Left Subthalamic Nucleus; 15mmAboveTarget: Left STN = 15 millimeters above target along stereotactic axis of left subthalamic nucleus; 75mmAboveTarget: Left STN = 75 millimeters above target along stereotactic axis of left subthalamic nucleus; RPPT = Right Posterior Pin Tip; RPPB = Right POsterior Pin Base; RPSS = Right Posterior Sphere Superior; RPSI = Right Posterior Sphere Inferior; RFPT = Right Frontal Pin Tip; RFPB = Right Frontal Pin Base; RFSS = Right Frontal Sphere Superior; RFSI = Right Frontal Sphere Inferior; LPPT = Left Posterior Pin Tip; LPPB = Left Posterior Pin Base; LPSS = Left Posterior Sphere Superior; LPSI = Left Posterior Sphere Inferior; LFSS = Left Frontal Sphere Superior; LFSI = Left Frontal Sphere Inferior; LFPT = Left Frontal Pin Tip; LFPB = Left Frontal Pin Base; Relecdelayed = Right Electrode Tip; Rscrew2 = Right Screw 2; RScrew1 = Right Screw 1; COA = Coherent On-Axis.

					Plane Intersection					
		3D Objects			Uncorrected (Not COA)				Corrected (COA)	
	AP	LAT	VERT	AP	LAT	VERT	90 degree	AP	LAT	VERT
AC	8.7	-0.8	-11.2	469.764	108.3856	-39.2966	5.462477	480.3335	-17.6189	-54.2145
PC	-18.8	-0.4	-11.6	466.7983	119.7995	-42.7545	5.469725	480.3335	-5.82648	-57.674
Midline	5.7	-2.3	41.9	467.3978	106.4912	50.25612	1.981663	480.3335	-18.8321	35.38136
Target: Left STN	-8.65882	-12.4276	-15.7972	473.3731	96.19302	-49.3322	6.141765	480.3335	-30.3306	-64.2548
15mmAboveTarget: Left STN	-2.35342	-20.1082	-4.56108	476.7589	81.2621	-29.4368	5.639001	480.3335	-45.6281	-44.3498
75mmAboveTarget: Left STN	22.86817	-50.8308	40.38327	489.8675	23.45393	47.59255	6.113697	480.3335	-104.856	32.7165
LFPB	89.4	-46.1	-5.6	494.4183	15.17863	-24.6257	7.853849	480.3335	-114.03	-39.5365
LFPT	74.6	-30	-4.1	486.9663	42.63178	-23.0215	6.709144	480.3335	-85.5838	-37.9315
LFSI	77.8	-58	-34.2	499.9613	0.097427	-70.6215	9.841432	480.3335	-130.034	-85.5544
LFSS	79.7	-54.5	-21.1	497.9987	4.887825	-49.7261	8.998951	480.3335	-124.898	-64.6489
LPPB	-92.9	-59.6	-2.7	487.4062	42.51261	-35.7165	7.134157	480.3335	-85.8133	-50.6326
LPPT	-75.5	-45.9	0.5	481.8148	62.24453	-27.2393	6.145061	480.3335	-65.3062	-42.1513
LPSI	-84	-65.3	-30.9	492.8405	28.97404	-91.2706	9.616863	480.3335	-100.3	-106.213
LPSS	-85	-64.2	-19.6	491.4975	31.27634	-68.7843	8.696894	480.3335	-97.7272	-83.7163
RFPB	87.8	49	-4.5	457.478	151.8248	-21.3616	4.317042	480.3335	27.52096	-36.2708
RFPT	72.5	33.5	-2.9	461.7529	135.8253	-20.1775	4.249801	480.3335	10.96017	-35.0861
RFSI	77.1	60.6	-38.4	453.5102	172.7025	-73.0388	6.979179	480.3335	48.7118	-87.9728
RFSS	79.3	56.7	-19.7	454.3771	166.1063	-44.6754	5.55964	480.3335	42.11733	-59.5959
RPPB	-94.1	48.5	-3.9	433.689	241.7109	-35.1035	7.051642	480.3335	120.5007	-50.0193
RPPT	-77	34.1	-1	443.0508	206.1689	-28.28	5.637865	480.3335	83.7471	-43.1926
RPSI	-86.6	58.3	-26.2	431.6135	254.3388	-76.4596	8.9513	480.3335	133.2336	-91.3953
RPSS	-87.4	57.5	-21.5	431.5886	253.3842	-67.6729	8.59737	480.3335	132.3184	-82.6044
Relecdelayed	-8.7	12.3	-16	462.5526	136.2857	-48.9331	5.653625	480.3335	11.1966	-63.8555
Rscrew2	42.1	40	41.4	453.9262	156.7134	48.17749	1.139342	480.3335	33.16547	33.30172
RScrew1	19.6	46.3	46.3	448.5058	175.8095	56.66613	1.773089	480.3335	53.0142	41.79445
Right Lateral Inferior Rod	0	140	-86	414.5516	327.3861	-158.305	14.19625	480.3335	208.2041	-173.28
Right Anterior Superior Rod	121.24	70	103	448.6916	165.9593	133.5392	3.562061	480.3335	43.45515	118.7045
Right Lateral Superior Rod	0	140	103	405.7873	323.4244	147.7571	10.03155	480.3335	206.6601	132.9292
Right Anterior Inferior Rod	121.24	70	-86	456.701	168.3064	-135.475	9.910497	480.3335	43.63674	-150.439
Left Lateral Inferior Rod	0	-140	-86	536.8558	-123.776	-179.637	17.69033	480.3335	-259.241	-194.623
Left Anterior Superior Rod	121.24	-70	103	500.4178	-26.5012	138.3634	9.221608	480.3335	-155.835	123.531
Left Lateral Superior Rod	0	-140	103	526.0007	-124.104	161.0163	14.06313	480.3335	-256.732	146.1948
Left Anterior Inferior Rod	121.24	-70	-86	509.1922	-25.4946	-143.216	13.22117	480.3335	-157.147	-158.184
Right Posterior Inferior Rod	-121.24	-70	-86	495.9658	32.44787	-217.707	14.77749	480.3335	-97.7591	-232.711
Right Posterior Superior Rod	-121.24	-70	103	483.5946	30.39441	184.6181	8.094983	480.3335	-96.5218	169.808
Left Posterior Superior Rod	-121.24	70	103	412.2495	296.2062	174.9906	9.486716	480.3335	178.698	160.1758
Left Posterior Inferior Rod	-121.24	70	-86	423.1228	300.8166	-202.163	15.09234	480.3335	180.3195	-217.159

Error propagation with PP

**Figure 10:**
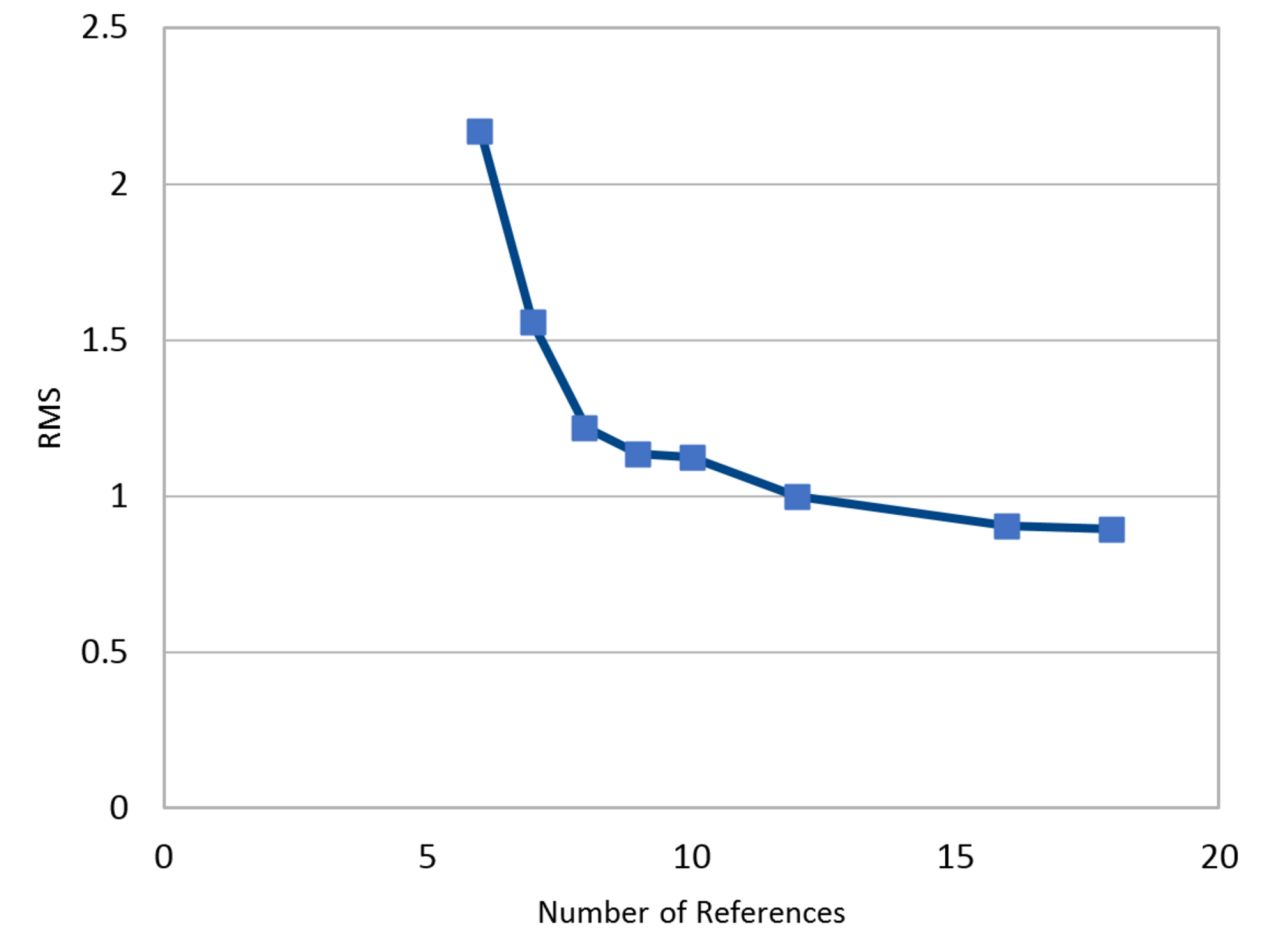
Monte Carlo simulation of 1 mm artificial error observing Root Mean Square (RMS) error in millimeters as a function of the number of references (points) over 1 million simulations. It is clear from the image that when six references are present, error propagation can be significant. Increasing the number of references to eight or more significantly decreases the propagation of an error. RMS = Root Mean Square Error

**Figure 11:**
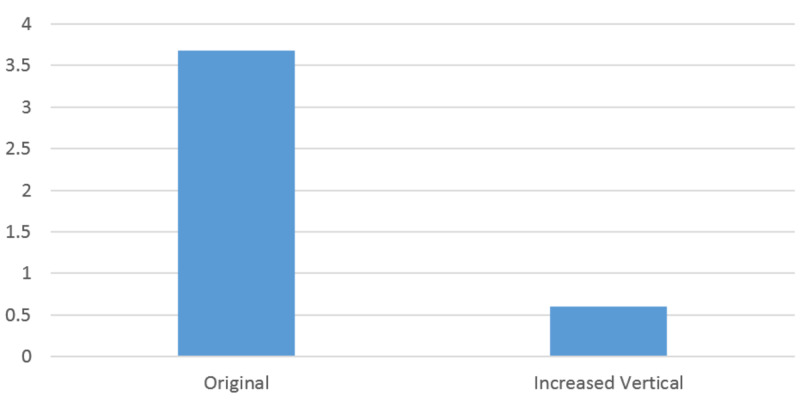
Influence of geometry on 3D Euclidean error propagation. In this image, an artificial error of 2 mm is introduced into AP, LAT, VERT, U or V values in either AP or LAT matrix formulations for PP. A worst-case 3D calculation is computed using AP and LAT PP formulation while fixing the fiducials at 8. The "Original" is the initial data from an actual case, whereas "Increased Vertical" has a 30 mm increase in vertical distance between points. The 3D error computation reduces from 3.69 mm to 0.6 mm from the "Original" to "Increased Vertical" datasets. While not a comprehensive study, this illustrates the impact of the imaging geometry on error propagation while using PP. AP = Antero-Posterior; LAT = Lateral; VERT = Vertical; U = horizontal screen coordinate; V = vertical screen coordinate; PP = Perspective Projection; Original = Initial Dataset from actual case; Increased Vertical = an artificial 30 mm increase in the vertical distance between points; Y-axis = 3D Euclidean error in millimeters.

While RT is computationally deterministic, PP utilizes a least squares optimization. Therefore, error propagation with PP is an important study. First, we evaluate automatic sphere detection on 3D images (CT) using a “center of mass” calculation and then evaluate potential errors. Hounsfield units and spherical size were used as thresholds for automatic sphere detection. This method would expedite acquisition of 3D points from CT imaging. Here, a comparison between this automation and manual selection is presented (Table 12). While this is an important step, error propagation in PP becomes relevant. We therefore introduced Monte Carlo simulation evaluating root mean square (RMS) as a function of the number of references when introducing random 1 mm errors into 1 million simulations (Figure 10). This reveals that error propagation is reduced by adding more data, which facilitates the least squares computation. In our physical setup, the vertical (VERT) distance between spheres had the least geometric variance. Fixing these points at 8, but increasing this VERT distance between them, causes a significant reduction in worst-case calculation of a 3D point when a 2-mm artificial error is introduced (Figure 11). This suggests that a wider geometric spread of fiducials reduces 3D error propagation while using PP.

**Table 12:** Comparison of manual selection versus automation for 3D fiducial spheres using a "center of mass" calculation. Single dimension error calculations are delta values and 3D errors are Euclidean distances. The average Euclidean error is 0.23 mm using this automation. Sphere = Spherical Fiducial identified automatically on CT; Delta AP = Error in Antero-Posterior dimension in millimeters; Delta LAT = Error in Lateral dimension in millimeters; Delta VERT = Error in Vertical dimension in millimeters; Euclidean Error = Total Euclidean 3D error.

Sphere	Delta AP	Delta LAT	Delta VERT	Euclidean Error
1	-0.05	0.1	-0.19	0.2205
2	-0.07	0	0.27	0.2789
3	-0.01	0.03	-0.01	0.0332
4	0.05	-0.02	0.16	0.1688
5	0.13	0.13	0.17	0.2504
6	0.11	0.2	0.13	0.2627
7	0.14	0.24	0.18	0.3311
8	0.22	0.17	-0.14	0.3113
Average	0.065	0.1063	0.0712	0.2321

## Discussion

X-ray imaging is widespread in various aspects of medicine. Single or multiple-plane computations, however, have not been widely utilized in standard X-rays imaging. While some efforts have been reported previously, herein, we have attempted a more comprehensive, usable, and illustrative approach [8-12]. We also explored various automations and error propagation. Further, enhanced computing power may facilitate these computations. Coincidentally, computer vision for 3D-2D rendering applies many similar principles to those used in X-ray imaging described above [7]. In computer vision, the task is generally to produce a 2D screen image from a dynamically changing 3D pose, but many modern applications include 3D-3D such as with virtual reality. Consideration of augmented reality also applies similar projection techniques superimposed on real-time analysis of images. Unlike our description for X-rays, the use of perspective projection with camera lenses may yield radial inhomogeneity (pincushion or barrel distortions) that require other steps or non-linear solutions.

Herein, we describe some of the mathematical principles of RT and PP applied to X-ray images and implement them in phantom and actual cases. Importantly, these methods have wide applications whenever planar images are obtained. Depending on the use case, simply increasing the number of reference points and the geometric spread ensures a quality result. However, RT depends more on knowledge of the imaging geometry. Also, application of scaling on the display image allows an enhanced understanding of the exposed geometry using PP. Three-dimensional imaging using CT or MRI images has largely overtaken 2D planar imaging when 3D positions are needed. However, CT/MRI incurs more expense and complexity, in addition to increased radiation (for CT) dose, without a significant improvement in resolution for a single target point. Further, the pixel size of an X-ray or fluoroscopy apparatus in our case were 0.125 mm and 0.388 mm, respectively, whereas the pixel size for 3D imaging is at best about 0.625 mm-0.8 mm for most current CT or MRI systems. Moreover, 3D imaging requires image fusion and introduces some artifacts by the nature of the acquisition and reconstruction techniques. Even though potentially millions of pixels are processed in 3D on CT/MRI, a single pixel displacement can result in a 0.625 mm error. In 2D imaging, for RT and PP, image point analysis is also required, but the 2D determination is at a higher resolution such that a single pixel displacement renders a 0.125 mm error. Therefore, this kind of point resolution to determine points in space, such as for deep brain stimulation (DBS) leads, can be sufficiently optimized using these simple X-ray techniques, which correlate well with preoperative CT/MRI imaging. In addition, as previously published, DBS lead rotation may be computed from projection X-ray data [2].

Important use cases for X-ray/fluoroscopy imaging include DBS, spine surgery, vascular interventions, non-vascular interventions, orthopedic interventions, radiosurgery and dental procedures as well as numerous non-medical applications or basic science laboratories, all of which can be enhanced with improved computing capacity [13 -16]. Because these systems can be designed using cost-effective materials, computational enhancements for these use cases could continue to grow. In addition, current X-ray and fluoroscopy technologies perform well with RT and PP, even though they were not designed for their use. Considering the mathematics and the physical nature of the X-ray systems, some assumptions should be considered carefully before applying RT or PP. Assumptions include that the system behaves like a Cartesian system, that the focal spot of the X-ray is a point source, that the X-ray detector is a plane, and that there is no significant scatter, or inhomogeneity in the image. We also assume X-ray and fluoroscopic imaging behave similarly. Some of these assumptions may not be completely accurate. For example, the focal spot of an X-ray can vary in size between different machines. Also, some fluoroscopic detectors may produce inhomogeneity. We also observed 2D images on some CT scanners, which were not a projection but rather a reconstruction. Therefore, care must be considered when applying these techniques to an imaging system.

## Conclusions

Ray tracing (RT) and perspective projection (PP) are useful tools for geometric imaging computation. RT or PP performs well for X-ray imaging analysis and may be used independently or together. PP can serve as an initial calibration for RT in a fixed setup when the geometry is unknown or difficult to measure. Because the pixel resolution of X-ray images is generally greater than CT/MRI, which also have reconstructive artifacts, these X-ray techniques offer excellent precision for point analysis. These tools have great importance in functional neurosurgery, such as with DBS, but can be extended to other medical or non-medical applications.
